# Lactosylceramide synthases encoded by *B4galt5* and *6* genes are pivotal for neuronal generation and myelin formation in mice

**DOI:** 10.1371/journal.pgen.1007545

**Published:** 2018-08-16

**Authors:** Toru Yoshihara, Hiroyuki Satake, Toshikazu Nishie, Nozomu Okino, Toshihisa Hatta, Hiroki Otani, Chie Naruse, Hiroshi Suzuki, Kazushi Sugihara, Eikichi Kamimura, Noriyo Tokuda, Keiko Furukawa, Koichi Fururkawa, Makoto Ito, Masahide Asano

**Affiliations:** 1 Institute of Laboratory Animals, Graduate School of Medicine, Kyoto University, Kyoto, Japan; 2 Division of Transgenic Animal Science, Advanced Science Research Center, Kanazawa University, Kanazawa, Japan; 3 Department of Bioscience and Biotechnology, Graduate School of Bioresource and Bioenvironmental Science, Kyushu University, Fukuoka, Japan; 4 Department of Molecular and Cell Structural Science, Kanazawa Medical University, Uchinada, Japan; 5 Department of Developmental Biology, Faculty of Medicine, Shimane University, Izumo, Japan; 6 Department of Biochemistry II, Nagoya University Graduate School of Medicine, Nagoya, Japan; 7 Department of Biomedical Sciences, Chubu University College of Life and Health Sciences, Kasugai, Japan; Istituto di Biochimica delle Proteine Consiglio Nazionale delle Ricerche, ITALY

## Abstract

It is uncertain which β4-galactosyltransferase (β4GalT; gene name, *B4galt*), β4GalT-5 and/or β4GalT-6, is responsible for the production of lactosylceramide (LacCer) synthase, which functions in the initial step of ganglioside biosynthesis. Here, we generated conditional *B4galt5* knockout (*B4galt5* cKO) mice, using *Nestin-Cre* mice, and crossed these with *B4galt6* KO mice to generate *B4galt5* and *6* double KO (DKO) mice in the central nervous system (CNS). LacCer synthase activity and major brain gangliosides were completely absent in brain homogenates from the DKO mice, although LacCer synthase activity was about half its normal level in *B4galt5* cKO mice and *B4galt6* KO mice. The DKO mice were born normally but they showed growth retardation and motor deficits at 2 weeks and died by 4 weeks of age. Histological analyses showed that myelin-associated proteins were rarely found localized in axons in the cerebral cortex, and axonal and myelin formation were remarkably impaired in the spinal cords of the DKO mice. Neuronal cells, differentiated from neurospheres that were prepared from the DKO mice, showed impairments in neurite outgrowth and branch formation, which can be explained by the fact that neurospheres from DKO mice could weakly interact with laminin due to lack of gangliosides, such as GM1a. Furthermore, the neurons were immature and perineuronal nets (PNNs) were poorly formed in DKO cerebral cortices. Our results indicate that LacCer synthase is encoded by *B4galt5* and *6* genes in the CNS, and that gangliosides are indispensable for neuronal maturation, PNN formation, and axonal and myelin formation.

## Author summary

Gangliosides are membrane-bound glycosphingolipids that contain sialic acid residues and are abundant in the mammalian nervous system, suggesting that they play pivotal roles in neural functions. We generated conditional β*4-galactosyltransferase-5* (*B4galt5*) knockout (KO) and double *B4galt5/B4galt6* KO (DKO) mice to completely ablate lactosylceramide (LacCer) synthase in the central nervous system (CNS). LacCer functions in the initial step of ganglioside biosynthesis. DKO mice were born normally but showed growth retardation and motor deficits at 2 weeks and died by 4 weeks of age. Myelin-associated proteins were rarely found localized in axons in the cerebral cortex, and axonal and myelin formation were remarkably impaired in the spinal cords of DKO mice. Neurospheres prepared from DKO mice could weakly interact with laminin, probably due to the lack of gangliosides in these mice. This defect might have caused the impaired neurite outgrowth in neuronal cells from DKO mice and poor formation of perineuronal nets (PNNs) with immature neurons in the cerebral cortices of DKO mice. Our results indicate pivotal roles for gangliosides in the CNS, including neuronal maturation, PNN formation, and axonal and myelin formation.

## Introduction

Gangliosides are membrane-bound glycosphingolipids (GSLs) that contain sialic acid residues. They are abundant in the mammalian nervous system, suggesting that they play pivotal roles in neural functions. Recent progress in the study of genetically engineered glycosyltransferases and relevant enzymes involved in the synthesis and modification of gangliosides in mice has elucidated various biological functions for gangliosides, especially in the nervous system [1]. For example, mice deficient in β4-N-acetyl-galactosaminyl transferase 1 (GM2/GD2 synthase), encoded by the *B4galnt1* gene, are viable and have a normal-life span, despite the lack of complex ganglio-series gangliosides and the accumulation of GM3 and GD3 [2,3]. They show dysmyelination and some axonal degeneration in the peripheral nervous system, similar to that seen in myelin-associated glycoprotein (*MAG*)-deficient mice [4]. In contrast, GM3 synthase-deficient mice lacking a- and b-series gangliosides have hearing impairments [5]. Since mice deficient in GD3 synthase, encoded by the *Siat8a* gene, show a relatively normal phenotype [6], double knockout (DKO) mice deficient in GM2/GD2 and GD3 synthases were previously generated. These DKO mice only produce GM3 and show a refractory skin lesion that is probably caused by reduced sensory function and peripheral nerve degeneration [7]. One group reported that DKO mice have a short-life span and experience fatal audiogenic seizures [8]. Reduced motor and sensory functions and emotional responses were also observed in these mice [9]. These results suggest that gangliosides other than GM3 play important roles in maintaining the integrity of the nervous system. To our surprise, mice expressing only GM3 are viable. To completely eliminate the synthesis of ganglio-series gangliosides, DKO mice deficient in GM2/GD2 and GM3 synthases were generated [10]. The authors showed that although pups are viable at birth, they develop rapid and profound neurodegeneration, and most of them die before 3 months of age.

Lactosylceramide (LacCer) is the starting point in the biosynthesis of all gangliosides. It is synthesized by LacCer synthase via the transfer of galactose from uridine diphosphate (UDP)-galactose to glucosylceramide (GlcCer); GlcCer is synthesized by GlcCer synthase via the transfer of glucose from UDP-glucose to ceramide (Cer) (S1 Fig). Although GlcCer synthase is encoded by a single gene, *Ugcg*, [11,12], LacCer synthase is probably encoded by several β*4-galactosyltransferase (B4galt)* genes. LacCer synthase was previously purified from rat brain homogenates and the corresponding cDNA was identified as *B4galt6* [13]. However, Chinese hamster ovary cells lacking *B4galt1* and *B4galt6* (Pro-5Lec20 cells), show LacCer synthase activity and produce LacCer, suggesting that other *B4galt* genes are responsible for the production of this enzyme [14,15]. Recently, we and another group generated *B4galt5* KO mice, which show early embryonic lethality [16,17]. LacCer synthase activity in *B4galt5*-deficient embryos and extra-embryonic endodermal (XEN) cells is reduced to about 10% of that found in wild-type embryos and heterozygous XEN cells, respectively. Our results suggest that LacCer synthase is mainly encoded by *B4galt5*, and not by *B4galt6*, at least in early mouse embryos [16]. However, it is still uncertain which of these two genes is responsible for the production of LacCer synthase in the mouse brain.

*Ugcg*-deficient mice show embryonic lethality [12], similar to *B4galt5* KO mice and conditional *Ugcg* knockout mice (*Ugcg* cKO; bred using *Nestin-Cre* mice) that are reported to exhibit severe neural defects after birth [18,19]. *Ugcg* cKO mice die around 3 weeks of age, show severe ataxia, and their peripheral nerve axons and myelin sheaths show mild degeneration. Neurite outgrowth and branching of primary cultured neuronal cells are also impaired in *Ugcg* cKO mice. However, the molecular mechanisms causing these neuronal defects have not yet been elucidated.

Since *B4galt5* KO mice show embryonic lethality, we generated *B4galt5* cKO mice by crossing *B4galt5*^*flox*^ (flox/flox) mice with *Nestin-Cre* transgenic (Tg) mice, in which Cre recombinase was expressed in neural precursor cells. *B4galt5* cKO mice were born normally, according to the Mendelian ratio, and developed to adulthood without any apparent abnormalities. Therefore, we next generated DKO mice for *B4galt5* and *6* in the CNS, by crossing *B4galt6* KO [20] and *B4galt5* cKO mice. DKO mice were born alive but showed growth retardation and motor deficits after 2 weeks of age and died by 4 weeks of age. LacCer synthase activity was completely absent in brain homogenates from DKO mice, although it was found to be at about half the normal levels in both the *B4galt5* cKO mice or *B4galt6* KO mice, clearly indicating that LacCer synthase is encoded by both *B4galt5* and *B4galt6* in the CNS. Our aim was to elucidate the cause of growth retardation and motor deficits in DKO mice and decipher the role of gangliosides in the CNS, including neuronal generation, and axonal and myelin formation using our DKO mice. Our results indicate pivotal roles for gangliosides in the CNS, including neuronal maturation, perineuronal net (PNN) formation, and axonal and myelin formation.

## Results

### Generation and study of *B4galt5* cKO mice

*B4galt5* cKO mice were generated by crossing *B4galt5*^*flox*^ mice and *Nestin-Cre* mice as described in detail in Materials and methods (S2 Fig). *B4galt5* cKO mice were born normally, according to the Mendelian ratio, and grew to adulthood without apparent abnormalities, in contrast to *B4galt5* KO mice that exhibit embryonic lethality [16,17]. *B4galt6* KO mice also appeared to be normal [20]. The expression levels of *B4galt5*, analyzed in whole-brain samples from *B4galt5* cKO mice at birth, 1 week, and 2 weeks of age using quantitative reverse transcriptase polymerase chain reaction (qRT-PCR), were reduced to less than 10% of those in samples from *B4galt5*^*flox*^ mice (control) at the same ages (S3A Fig). When the adult mouse brain was divided into 10 sub-regions, the expression levels of *B4galt5* in the olfactory bulb, striatum, cerebral cortex, thalamus, hippocampus, and cerebellum of *B4galt5* cKO mice were also reduced to less than 10% of the levels in the respective regions of *B4galt5*^*flox*^ mice, but over 50% of the control levels were detected in the hypothalamus, pons, midbrain, and medulla oblongata, probably because non-neural cells not expressing nestin were abundant in these brain regions (S3B Fig). The expression levels of the *B4galt6* gene were not affected in *B4galt5* cKO brains (S3C and S3D Fig).

**Fig 1:**
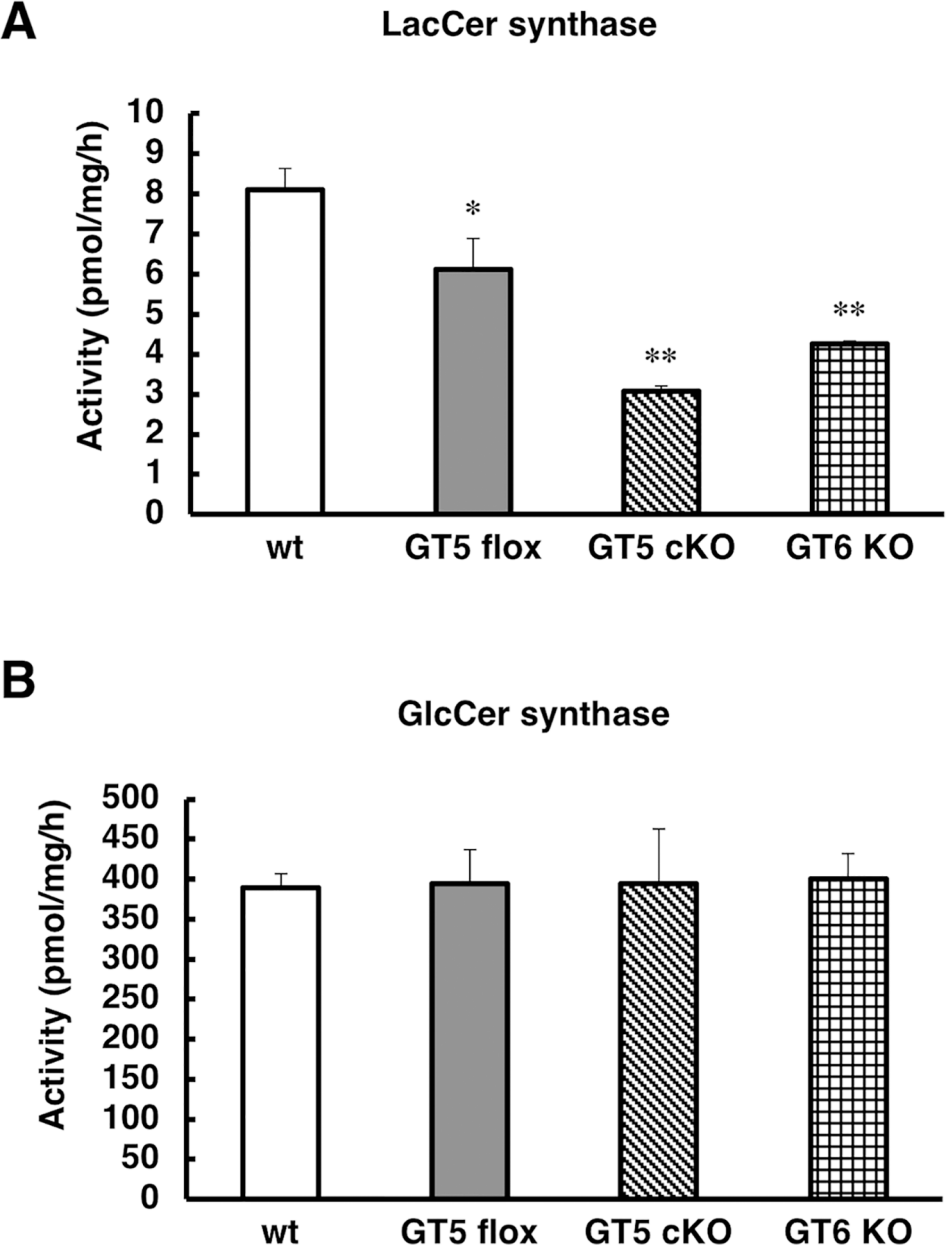
LacCer and GlcCer synthase activity in a single KO mice. (A) LacCer synthase activity was measured in brain homogenates from wild-type (wt), *B4galt5*^*flox*^ (GT5 flox), *B4galt5* conditional knockout (GT5 cKO), and *B4galt6* KO (GT6 KO) mice (n = 3 per genotype) at 3 weeks of age. (B) GlcCer synthase activity was measured in brain homogenates from the same mice in A (n = 3 per genotype). *, *p*<0.05; **, *p*<0.01.

We measured LacCer and GlcCer synthase activity in brain homogenates from *B4galt5* cKO and *B4galt6* KO mice using high-performance liquid chromatography (HPLC). As shown in Fig 1A and 1B, LacCer synthase activity remained at 38% and 52% of the wild-type levels in *B4galt5* cKO and in *B4galt6* KO mice, respectively, although GlcCer synthase activity was at almost the same level in all mice. LacCer synthase activity was slightly lower in *B4galt5*^*flox*^ than in wild-type mice. These results suggest that LacCer synthase is derived from both *B4galt5* and *6* genes in the brain.

### Generation of *B4galt5* and *B4galt6* DKO mice

**Fig 2:**
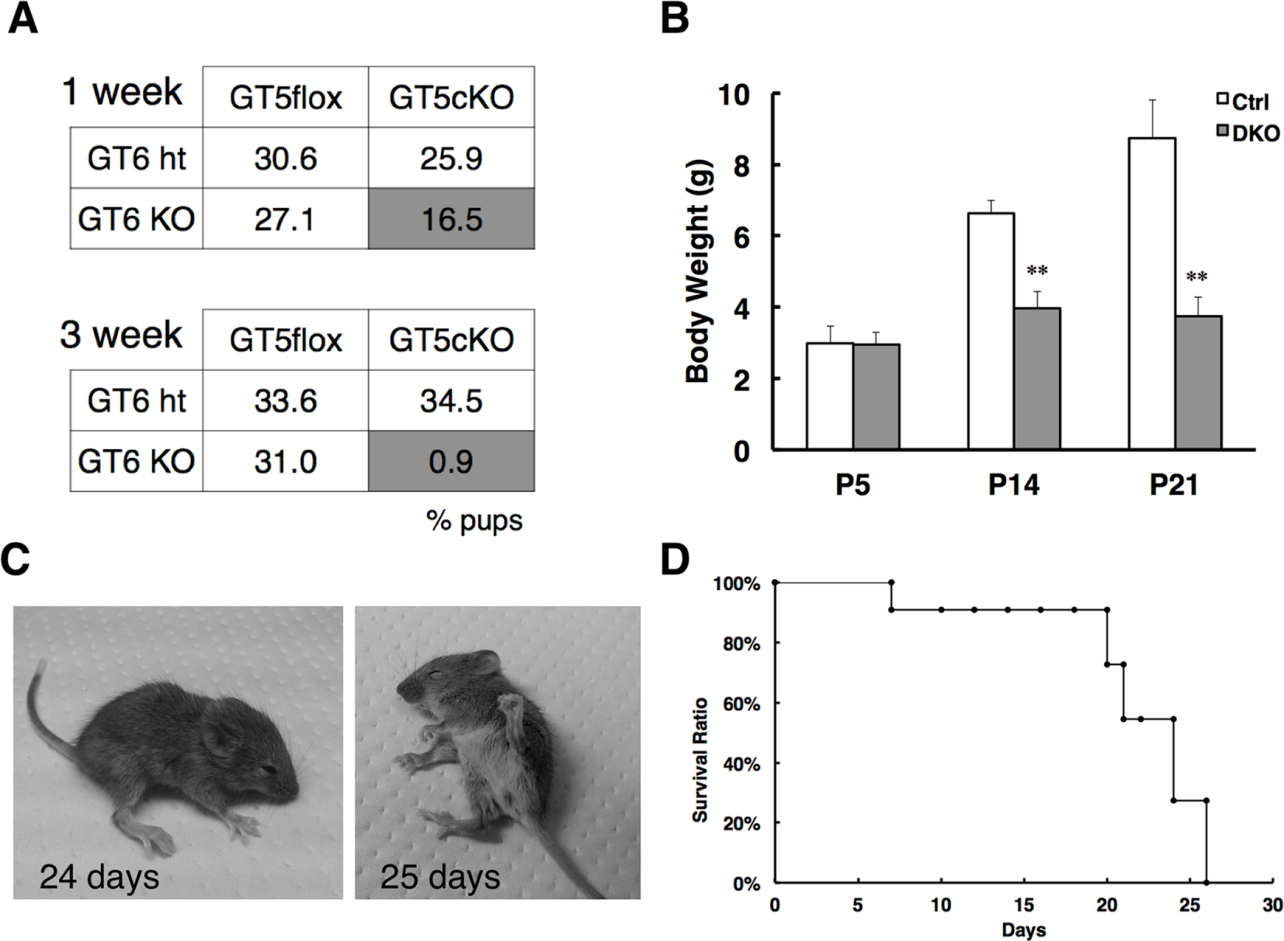
Growth retardation and early lethality in DKO mice. (A) Survival ratios of double knockout (DKO) mice at 1 and 3 weeks of age, generated from breeding of *B4galt5*^*flox*^(flox/flox)/*Nestin-Cre*(+/+)/*B4galt6*(-/-) female mice and *B4galt5*^*flox*^(flox/flox)/*Nestin-Cre*(tg/+)/*B4galt6*(+/-) male mice. The total number of pups at 1 week of age was n = 85 and at 3 weeks n = 113. (B) Body weights of juvenile mice of each genotype at postnatal day 5 (P5), 14 (P14), and 21 (P21). White bar, *B4galt5*^*flox*^*/B4galt6* ht (P5, n = 4; P14, n = 8; P21, n = 8); black bar, *B4galt5* cKO/*B4galt6* KO (P5, n = 4; P14, n = 8; P21, n = 8). (C) The gross appearances of individual DKO mice at 24 and 25 days of age. (D) Survival curve of DKO mice (n = 7) before weaning. **, *p*<0.01.

We intercrossed *B4galt5*(flox/flox)/*Nestin-Cre*(+/+)/*B4galt6*(-/-) female mice with *B4galt5*(flox/flox)/*Nestin-Cre*(tg/+)/*B4galt6*(+/-) male mice to produce *B4galt5*(flox/flox)/*Nestin-Cre*(tg/+)/*B4galt6*(-/-) DKO mice, expected to be 25% of the offspring. At 1-week-old, DKO mice (16.5% of a total of 85 pups) were alive, while at 3-weeks-old fewer DKO mice (0.9% of a total of 113 pups) were alive, suggesting that these mice died between 1 and 3 weeks of age (Fig 2A). DKO mice appeared normal at birth and their body weight at 5 days of age was comparable with that of control mice. These results suggest that the DKO mice developed normally during embryogenesis and perinatal stage. However, they showed growth retardation (Fig 2B) and motor deficits with hindlimb dysfunction at 2 weeks of age, and they all died by 4 weeks of age (Fig 2C and 2D). We thought that the mice might have difficulty in consuming food in the usual mouse cages, because they showed severe hindlimb dysfunction. Thus, we compared the body weight of each genotype at 14 and 21 days of age. We found that the body weight of *B4galt5* cKO/*B4galt6* heterozygous (ht) mice, which had only a single copy of LacCer synthase genes in neural tissues, was slightly, but significantly lower than the weight of *B4galt5*^*flox*^*/B4galt6* ht and *B4galt5*^*flox*^*/B4galt6* KO mice (S4 Fig). These observations suggest that the *B4galt5* cKO/*B4galt6* ht mice might be used as a potential human rare disease model, characterized by residual low LacCer synthase activity.

**Fig 3:**
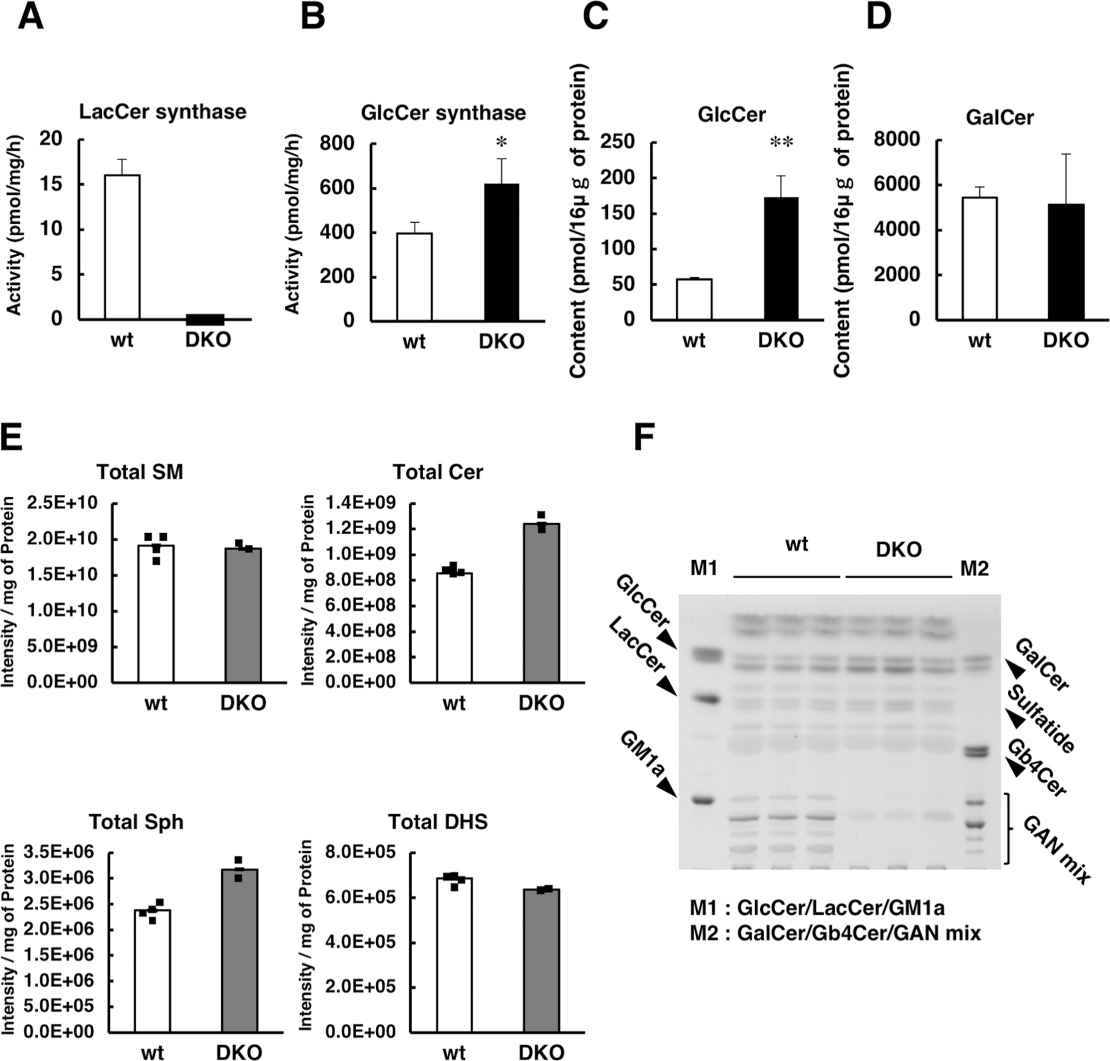
LacCer and GlcCer synthase activity and sphingolipid analysis in DKO mice. (A) LacCer synthase activity was measured in brain homogenates from wild-type (wt) and *B4galt5* and *6* double KO (DKO) mice (n = 3 per genotype) at 3 weeks of age. (B) GlcCer synthase activity was measured in brain homogenates from the same mice in A (n = 3 per genotype). The levels of GlcCer (C) and GalCer (D) in brain homogenates of the same mice as in (A) and (B) were measured (n = 3 per genotype). (E) The levels of total SM, Cer, Sph, and DHS in brain homogenates from wt and DKO mice (wt, n = 4; DKO, n = 2) at 3 weeks of age. Note that small squares on the bar graph indicate the individual values of each sample. (F) GSLs extracted from brain homogenates from wt and DKO mice at 3 weeks of age were separated by high performance thin layer chromatography (n = 3 per genotype). M1 and M2, standard GSLs are indicated. *, *p*<0.05; **, *p*<0.01.

LacCer synthase activity was completely absent in brain homogenates from DKO mice (Fig 3A), whereas residual LacCer synthase activities were detected in single KO mice (Fig 1A). These results clearly indicate that LacCer synthase is encoded by both *B4galt5* and *B4galt6* genes, and that no other *B4galt* gene is involved in LacCer synthesis in the CNS. GlcCer synthase activity was increased 1.5-fold compared to that in wild-type mice (Fig 3B), whereas *Ugcg* (GlcCer synthase gene) mRNA levels were comparable between genotypes (S3E Fig), probably due to the accumulation of GlcCer synthase. We next measured the levels of GlcCer and GalCer (a product generated from Cer by GalCer synthase) in brain homogenates (Fig 3C and 3D). GlcCer levels were about three-fold higher in DKO than in wild-type mice, whereas GalCer levels were comparable between genotypes, suggesting that GlcCer accumulated in the brains of DKO mice owing to a lack of LacCer synthase. Liquid chromatography-electrospray ionization mass spectrometry (LC-ESI MS) analysis showed that the levels of Cer and sphingosine (Sph), upstream of the synthetic pathway of GlcCer, were slightly higher in DKO than in wild-type mice. In contrast, the levels of sphingomyelin (SM) and dihydrosphingosine (DHS), two sphingolipids downstream of Cer and upstream of Sph, respectively, were comparable between genotypes (Fig 3E). Detailed analysis of the levels of individual sphingolipid molecules, i.e., Cer, SM, Sph and DHS is shown in S2 Table. Since LacCer is the starting point in ganglioside biosynthesis, GSLs in brain homogenates were analyzed by thin-layer chromatography (TLC). Gangliosides, including GM1a, GD1a, and GT1b, were not detected in the brains of DKO mice (Fig 3F), although they were detected at almost the same level among wild-type, *B4galt5*^*flox*^, *B4galt5* cKO, and *B4galt6* KO mice (S5 Fig). These results indicate that gangliosides were absent only in the brains of DKO mice.

### Increased immature neurons in the cerebral cortices of DKO mice

**Fig 4:**
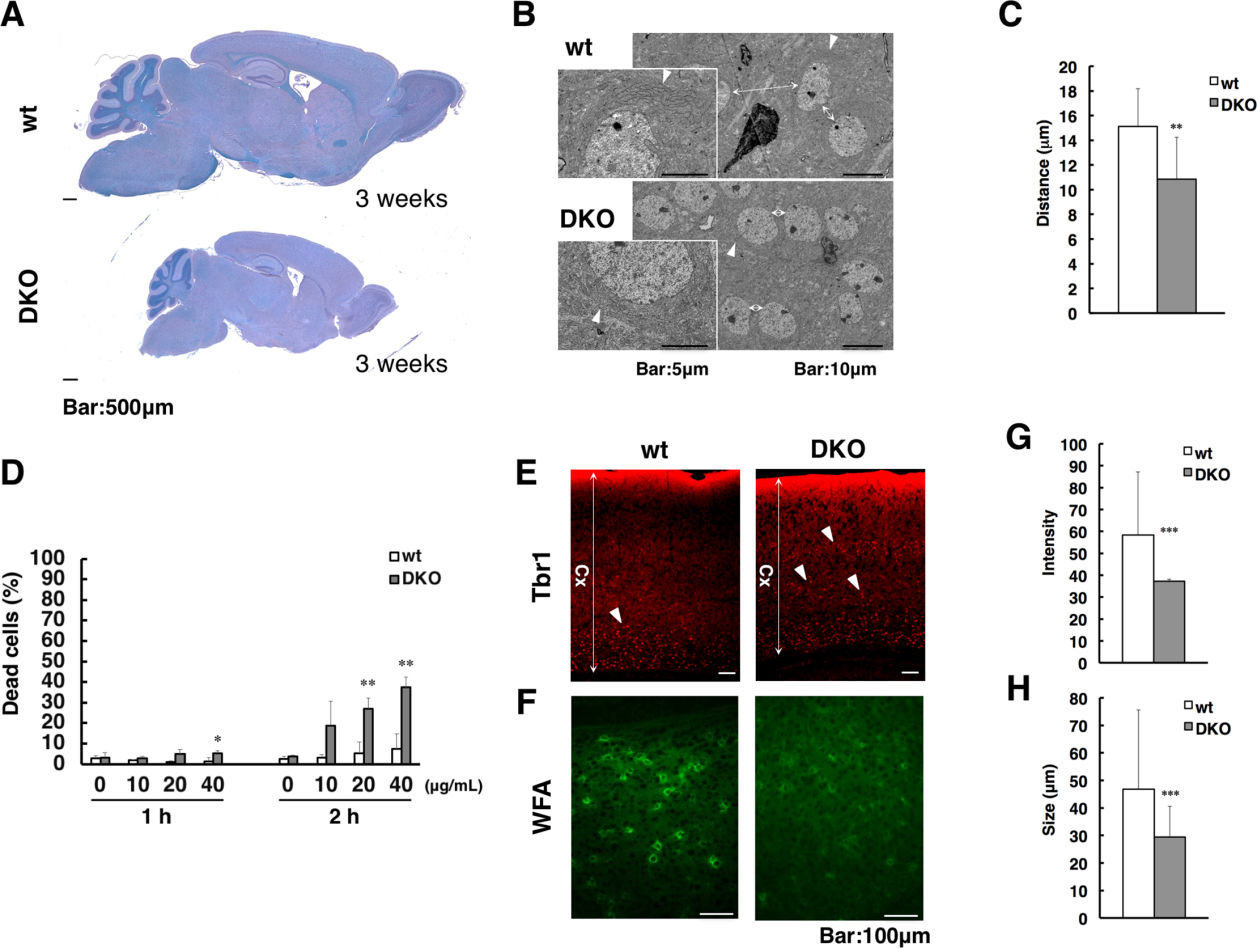
Histological analysis of whole brains and cerebral cortices. (A) Klüver-Barrera (KB) staining of sagittal brain sections from wild-type (wt) (upper) and double knockout (DKO; lower) mice at 3 weeks of age. (B) TEM analysis of the cerebral cortices of wt and DKO mice at 3 weeks of age. Left-hand pictures show a higher magnification of the nuclei indicated by arrowheads in the right-hand pictures. Arrowheads: rough ER; arrows: distance to adjacent nuclei. (C) The distance to adjacent nuclei was measured in the cerebral cortices of wt (n = 20) and DKO (n = 30) mice. (D) The ratio of dead cells in neurosphere cultures prepared from wt and DKO mice at 1 h (left) and 2 h (right) after treatment with cycloheximide at the indicated concentrations. (E) Immunohistochemical staining for Tbr1, an immature neuronal marker, on cerebral cortex sections prepared from wt and DKO mice at 3 weeks of age. Arrowheads: Tbr1-positive cells. (F) WFA lectin staining of cerebral cortex sections from wt and DKO mice at 3 weeks of age. Peak fluorescence intensity (G) and maximum area surrounded by WFA positive signals (H) in the cerebral cortices of wt (20 areas × 3 mice) and DKO (20 areas × 3 mice) mice are shown. **, *p*<0.01; ***, *p*<0.001.

We examined the brains of DKO mice histologically. The brain of the DKO mice at 3 weeks of age was smaller than those of wild-type mice in proportion to their body sizes, but its gross morphology appeared normal (Fig 4A). Transmission electron microscopic (TEM) analysis revealed that the rough endoplasmic reticulum (ER) was poorly formed around the nucleus in neuronal cells in the cerebral cortices of the DKO mice (Fig 4B), suggesting that these neuronal cells had a deficiency in protein synthesis. Furthermore, the nuclei of DKO neuronal cells were close to each other because of their unexpanded cytoplasm (Fig 4B and 4C). These TEM observations also suggested that the neurons in the DKO mice were in an immature state. To evaluate protein synthesis ability of DKO neural cells, we prepared neurospheres from the ganglionic eminence (GE) of embryonic day (E) 14.5 embryos and examined the effect of the protein synthesis inhibitor, cycloheximide. The ratio of dead cells increased more in DKO than in wild-type neurospheres treated with cycloheximide, in a dose-dependent manner. Significant differences were detected after one- and two-hour cycloheximide treatment at 40 μg/mL and at 20–40 μg/mL, respectively, strongly suggesting that DKO neurospheres exhibit deficient protein synthesis (Fig 4D). We further examined the expression of an immature neuronal marker, T-box brain gene 1 (Tbr1) [21], in the cerebral cortices at 3 weeks of age, by performing immunohistochemical staining. Tbr1-positive immature neurons were almost restricted in layer VI in wild-type cerebral cortices, whereas they extended towards upper layers in DKO cerebral cortices (Fig 4E). These results strongly suggest that neurons in the upper layers of DKO cerebral cortices were still in an immature state.

Since neurons in the cerebral cortices of the DKO mice at 3 weeks of age remained at a premature stage, we hypothesized that the PNNs might not be properly formed in these mice. We thus examined the formation of PNNs in the cerebral cortex using *Wisteria floribunda* agglutinin (WFA) lectin staining, which specifically marks PNNs (Fig 4F). The peak fluorescence intensity of WFA staining was much weaker and the maximum area of PNNs was significantly smaller in the cerebral cortices of DKO than of wild-type mice (Fig 4G and 4H). These results indicate that the PNNs were not properly formed in the cerebral cortices of DKO mice. Taken together, these results strongly suggest that the neurons in the cerebral cortices of DKO mice were relatively immature.

### Proliferation, adhesion to extracellular matrices (ECMs), and neurite outgrowth in neurosphere cultures

**Fig 5:**
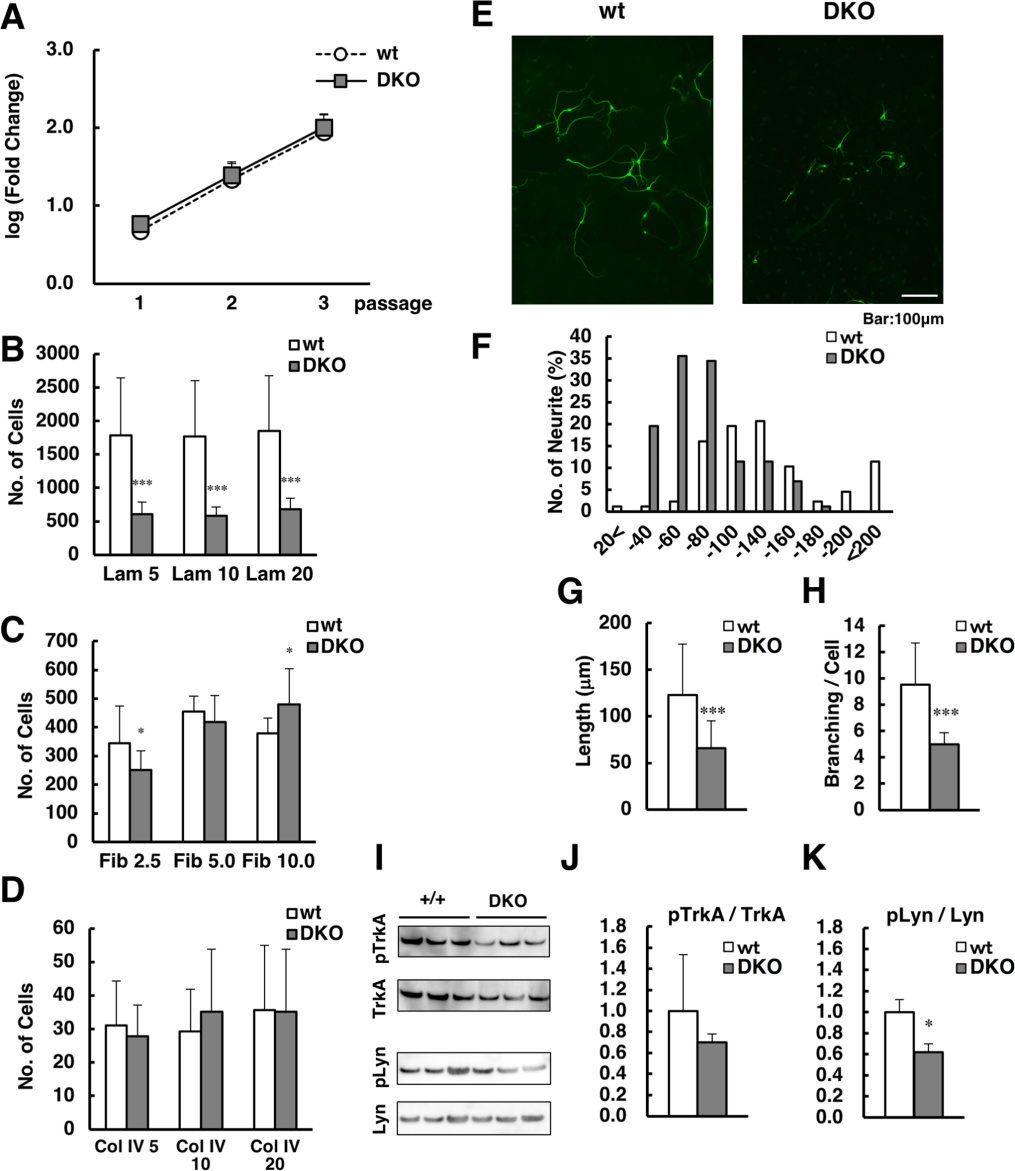
Proliferation, adhesion to ECMs, and neurite outgrowth in neurosphere cultures. (A) Proliferation in neurosphere cultures prepared from wild-type (wt, n = 3) and double knockout (DKO, n = 3) mice, from passage 1 to passage 3. (B–D) Cell number quantification in neurospheres prepared from wt (n = 3) and DKO (n = 3) mice that adhered to culture dishes coated with laminin (B), fibronectin (C), and collagen IV (D) at the concentrations indicated. (E) Neuronal cells, stained using anti-βIII-tubulin, differentiated from neurospheres from wt (upper) and DKO (lower) mice. Note that the neurite/axon length and number of branches were lower in DKO neuronal cells than in wt cells. (F) Distribution of the longest neurite length from an individual wt and DKO cell. (G) Mean length of the longest neurite of wt (n = 87) and DKO (n = 101) neuronal cells. (H) Mean of the total number of neuronal branching points per cell in wt (n = 23) and DKO (n = 20) neuronal cells. (I) Western blot analysis of neurospheres prepared from wt (n = 3) and DKO (n = 3) mice using anti-pTrkA (Y490ph), TrkA, pLyn (Y396ph), and Lyn antibodies. (J, K) The relative levels of pTrkA/TrkA (J) and pLyn/Lyn (K) of wt and DKO neurospheres are shown. The protein levels in wt neurospheres were normalized to 1. *, *p*<0.05; ***, *p*<0.001.

To further characterize DKO neurons, we prepared neurospheres from the GE of E14.5 embryos. The proliferation of neurospheres from DKO mice was comparable to that of neurospheres from wild-type mice, indicating no impairment (Fig 5A). We next examined the ability of neurospheres, dissociated to a single cell, to adhere to several ECMs, including laminin, collagen, and fibronectin. Although cell adhesion to fibronectin and collagen IV was comparable between neurospheres from wild-type and DKO mice, cell adhesion to laminin was markedly reduced in DKO neurospheres (Fig 5B, 5C and 5D). Since laminin is reported to bind directly to GM1a and activate neurite outgrowth in lipid rafts [22], we further examined neurite outgrowth and branch formation in these cells. As shown in Fig 5E, neurite length and branch formation were apparently reduced in neurospheres from DKO mice. While neurite length was between 80 to 160 μm in neurospheres from wild-type mice, it was mainly between 40 to 80 μm in neurospheres from DKO mice (Fig 5F), and the average neurite length in neurospheres from DKO mice was about half of that in neurospheres from wild-type mice (Fig 5G). The number of branching points in neurites from DKO mice was reduced to about half of that in neurites from wild-type mice (Fig 5H). These results indicate that neurite outgrowth and branch formation were severely impaired in neurospheres from DKO mice. To further examine the signaling cascade regulating ganglioside-laminin interaction, we examined the phosphorylation levels of tyrosine kinase receptor A (TrkA) and tyrosine kinase Lyn in neurospheres by western blot (Fig 5I). The ratio of pTrkA (Y490ph)/TrkA tended to be lower and that of pLyn (Y396ph)/Lyn was significantly lower in DKO than in wild-type neurospheres (Fig 5J and 5K). This suggests that the signaling cascade downstream of TrkA was reduced. These defects in neurospheres from DKO mice might result in the poor formation of PNNs.

### Impaired axonal and myelin formation in DKO mice

**Fig 6:**
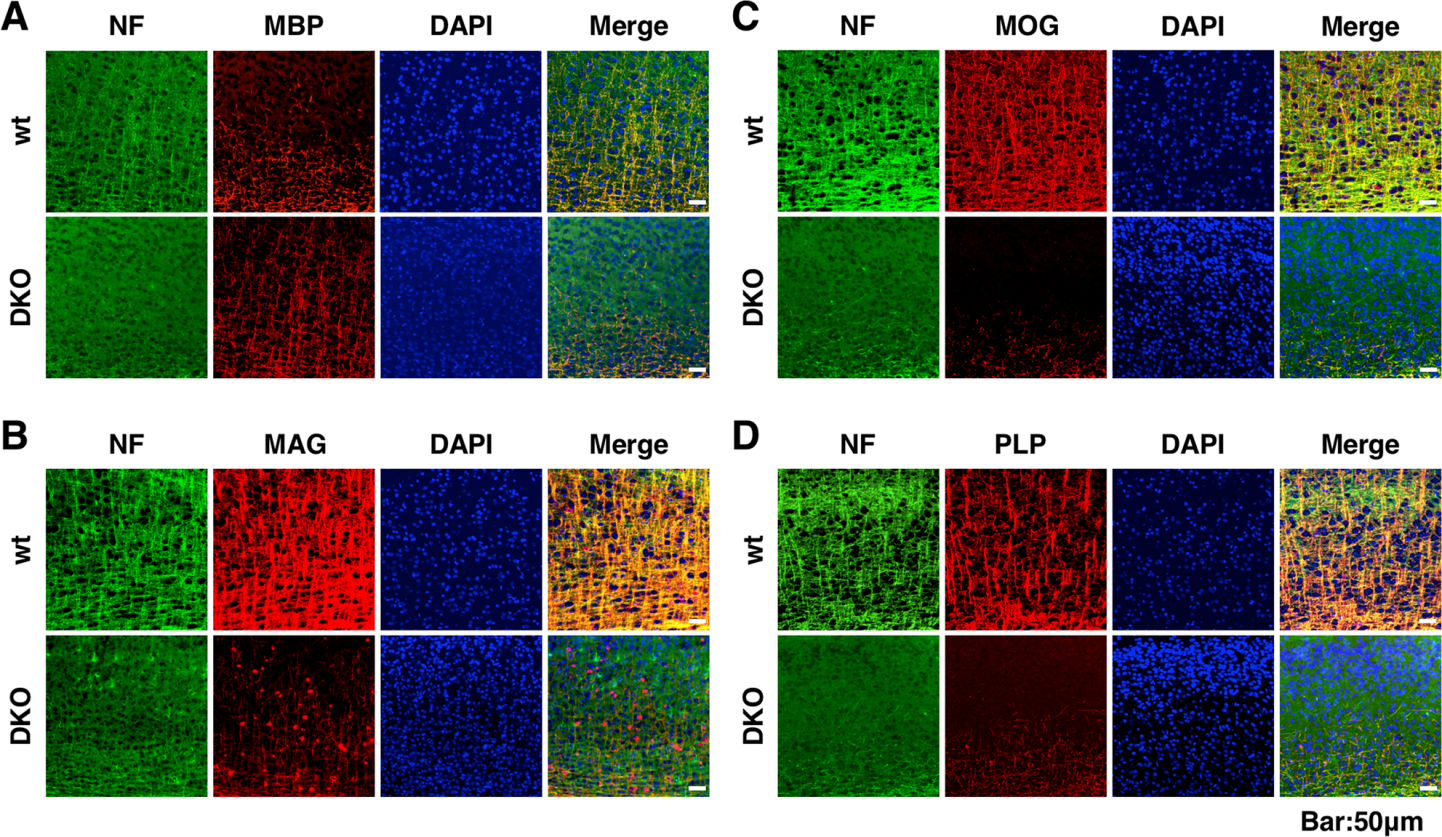
Immunohistochemical analysis of myelin-associated proteins and neurofilament (NF) in the cerebral cortex. (A–D) Immunohistochemical staining for MBP (A, red), MAG (B, red), MOG (C, red), and PLP (D, red) merged with NF (green) and DAPI (blue) in the cerebral cortices of wild-type (wt) and double knockout (DKO) mice at 3 weeks of age.

Next, we examined the co-localization of myelin-associated proteins, including myelin basic protein (MBP), MAG, myelin-oligodendrocyte glycoprotein (MOG), and proteolipid protein (PLP) with neurofilament (NF) by immunohistochemical staining in the cerebral cortex (Fig 6). In the wild-type cerebral cortex, these myelin-associated proteins were all associated with NF, indicating that they interacted with axons. However, NF signals were thinner and weaker in DKO than in wild-type mice. Furthermore, MOG and PLP signals were barely detected and poorly co-localized with NF in the cerebral cortices of DKO mice (Fig 6C and 6D), whereas MBP signals in DKO mice appeared to be the same as in wild-type mice, although they were poorly co-localized with NF (Fig 6A). MAG signals were also weak but showed a dot pattern and were not associated with NF in DKO mice (Fig 6B). Western blot analysis showed that the levels of MAG and MOG were lower in brain homogenates of DKO than of wild-type mice, whereas the levels of MBP and PLP were comparable between genotypes (S6 Fig). These results suggest that apart from the impaired localization of these myelin-associated proteins in DKO mice, the production of MAG and MOG was also reduced.

**Fig 7:**
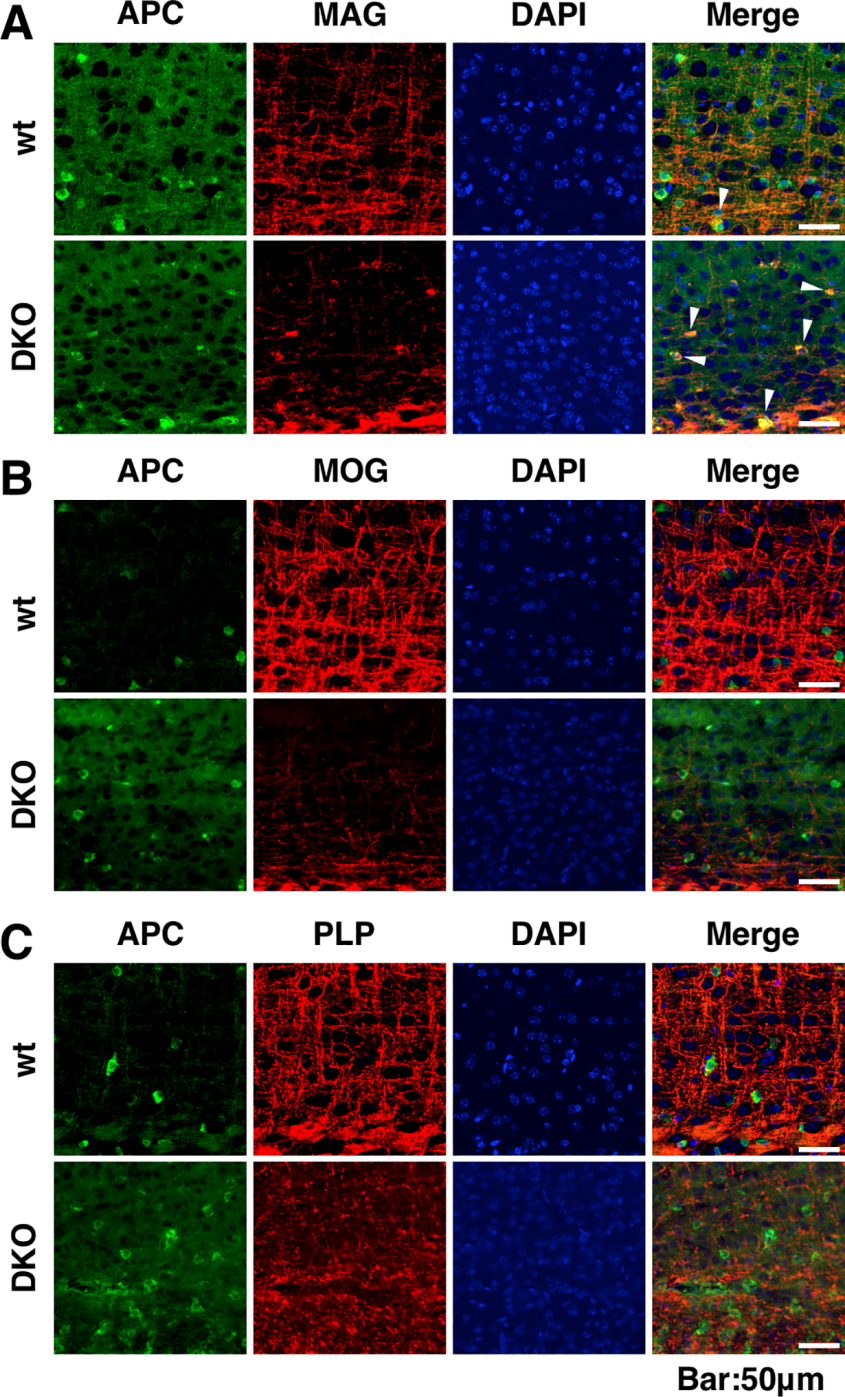
Immunohistochemical analysis of myelin-associated proteins and adenomatous polyposis coli (APC) in the cerebral cortex. (A–C) Immunohistochemical staining for MAG (A, red), MOG (B, red), and PLP (C, red) merged with APC (green) and DAPI (blue) in the cerebral cortices of wild-type (wt) and double knockout (DKO) mice at 3 weeks of age.

To further examine the dot pattern of MAG localization, we conducted double immunohistochemical staining for MAG, MOG, or PLP with adenomatous polyposis coli (APC), a marker for oligodendrocyte cell bodies. As shown in Fig 7A, the dotted MAG signals were associated with APC (arrowheads), suggesting that MAG accumulated in the oligodendrocyte cell bodies. On the other hand, MOG and PLP were not associated with APC (Fig 7B and 7C). These results suggest that MAG accumulated in oligodendrocyte cell bodies, whereas MOG and PLP were diffuse and rarely localized in the myelin sheath in the cerebral cortices of DKO mice.

**Fig 8:**
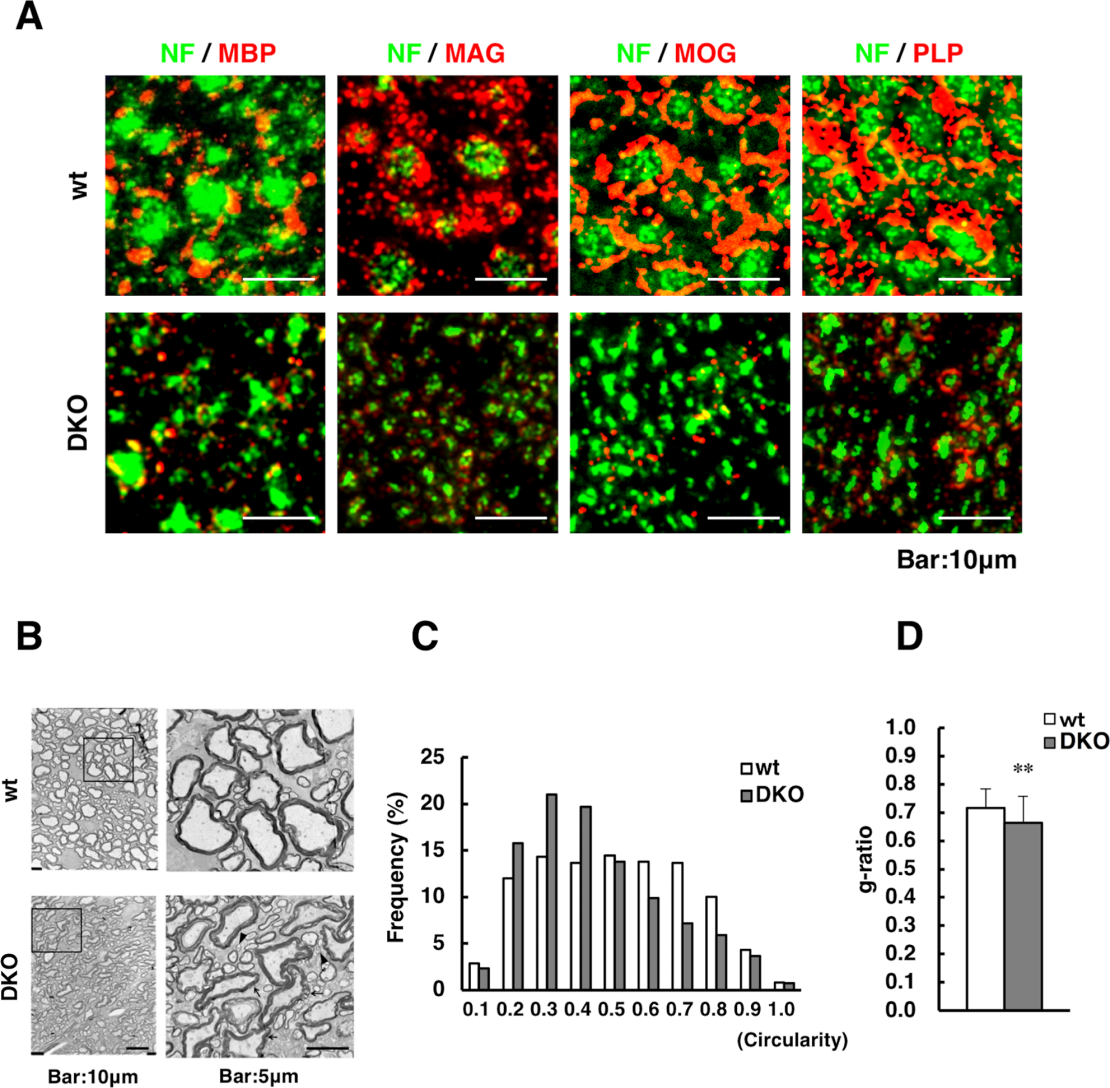
Immunohistochemical and TEM analysis in the spinal cord. (A) Immunohistochemical staining for MBP, MAG, MOG, and PLP (red) merged with NF (green) in the spinal cords of wild-type (wt) and double knockout (DKO) mice at 3 weeks of age. (B) TEM analysis of the spinal cords of wt (upper) and DKO (lower) mice at 3 weeks of age. Right-hand pictures show a higher magnification of the squares in the left-hand pictures. Arrowheads: small axons without a myelin sheath; arrows: large axons with low circularity. (C) Frequency of axon circularity in wt (n = 1930) and DKO (n = 2120) mice. (D) The g-ratio (axon diameter/myelinated fiber diameter) of large axons of wt (n = 130) and DKO mice (n = 95) was measured. **, *p*<0.01.

Next, we examined the localization of MBP, MAG, MOG, and PLP in the spinal cords of DKO mice (Fig 8A). In the spinal cords of wild-type mice, myelin-associated proteins were wound around thick axons that stained with NF. However, axons were thin and myelin-associated proteins were rarely found around axons in the spinal cords of DKO mice, although PLP was detected to some extent around thin axons in these mice.

TEM analysis of the spinal cord showed that the axons were markedly hypoplastic, with lower axon circularity in DKO than in wild-type mice (Fig 8B and 8C). Small axons without myelin sheaths were more abundant in DKO than in wild-type mice (arrowheads, Fig 8B). Some of the large axons in DKO mice had thin or less packed myelin sheaths (arrows, Fig 8B). The relative distribution of the myelin and axon area of large axons was analyzed by the g-ratio [23]. The g-ratio was significantly lower in DKO than in wild-type axons, suggesting that DKO axons were wrapped loosely with low-density myelin sheaths (Fig 8D). This impaired axonal and myelin formation in the spinal cord might account for the motor deficits observed in DKO mice.

## Discussion

### LacCer synthase is encoded by *B4galt5* and *B4galt6* genes in the CNS

We have previously reported that *B4galt5* KO mice show embryonic lethality and that *B4galt6* is expressed normally in *B4galt5*-deficient embryos, suggesting that *B4galt6* cannot compensate for the *B4galt5* deficiency in early mouse embryos. Consistently, residual LacCer synthase activity in *B4galt5*-deficient embryos and XEN cells is about 10% of that in wild-type embryos and *B4galt5* +/- XEN cells, respectively [16]. These results suggest that the contribution of *B4galt6* gene to LacCer synthase production and ultimately activity in early mouse embryos is 10% or lower. However, when LacCer synthase was purified from rat brain homogenates, the corresponding cDNA identified was *B4galt6* [13]. It is uncertain whether this difference is due to tissue differences or species differences. To clarify the gene(s) responsible for production of LacCer synthase in the CNS, we generated neural cell specific *B4galt5* cKO mice and DKO mice deficient in both *B4galt5* and *B4galt6* genes in the CNS. LacCer synthase activity in the brain remained at 38% in *B4galt5* cKO mice and 52% in *B4galt6* KO mice compared to the activity in wild-type mice, while it was completely absent in DKO mice. Consistently, gangliosides were not detected in the brains of DKO mice, although they were detected in the brains of single KO mice. These results clearly indicate that both *B4galt5* and *B4galt6* genes are responsible for LacCer synthase activity in the brain, although we have not examined LacCer synthase in brains from other species, such as rats. Taken together, these results suggest that, at least in mice, β4GalT-5 and -6 are the main LacCer synthases in the CNS, whereas β4GalT-5 is the main LacCer synthase in early embryos.

### Motor deficits and early lethality in DKO mice

Each ganglioside is thought to have an independent role in the nervous system given that various gangliosides are abundantly produced in the mammalian brain. KO mice deficient in various ganglioside synthase genes have been reported to have various defects in the nervous system, as described in the introduction [1]. With regard to the ganglioside biosynthetic pathway, mice deficient in upstream (GlcCer) or downstream synthases (GM2/GD2 and GM3) of LacCer synthase have been generated and analyzed; here, we present evidence on mice deficient in LacCer synthase in the CNS. The phenotypes of DKO mice deficient in GM2/GD2 and GM3 synthases, lacking all ganglio-series gangliosides, appear to be different from the phenotype of our DKO mice. These mice eventually die at around 4–5 months of age and present with hind limb weakness, ataxia, and tremors. They also exhibit vacuolization in the spinal cord and cerebellar white matter, as well as apoptotic cells in the cerebral cortex at 2.5 months of age, indicating severe neurodegeneration [10]. In contrast, our DKO mice die within 4 weeks of age, before these neurodegeneration phenotypes are detected. On the other hand, *Ugcg* (GlcCer synthase) CNS cKO mice show motor deficits and die at around 3 weeks of age. Neuronal axons and myelin are degenerated, and neurite length and number of branching points are reduced, as seen in primary neuronal cultures prepared from *Ugcg* cKO mice. Therefore, these mice show similar phenotypes to our DKO mice [18]. However, the molecular mechanisms involved in the neural defects induced by complete ganglioside-deficiency in the CNS remain to be elucidated.

Although the above-mentioned mutant mouse lines completely lack ganglio-series gangliosides, including the abundant ones in neurons, GM1a, GD1a, GD1b, and GT1b (S1 Fig), the accumulated upstream sphingolipids are different among different genotypes. While LacCer and SM3, direct upstream of GM3 and SM2a, accumulate in GM2/GD2 and GM3 DKO mice [10], SM, but not Cer, accumulates in GlcCer synthase-deficient mice [18]. On the other hand, we found a three-fold accumulation of GlcCer and a slight accumulation of Cer and Sph, but not SM and DHS, in our LacCer synthase-deficient mice. Therefore, the similar phenotypes among these three mutant mice seem to be caused by the complete lack of ganglio-series gangliosides, while the different phenotypes might arise from differences in the accumulated sphingolipids and GSLs. The accumulation of GlcCer in macrophages present in various organs, including the liver, spleen, and bone marrow, is known as Gaucher disease [24], which is caused by mutations in the gene encoding for glucocerebrosidase, an enzyme that hydrolyzes GlcCer. Further studies will be necessary to elucidate the relationship between Gaucher disease and LacCer synthase-deficiency.

### Impaired axonal and myelin formation in DKO mice

Gangliosides act as receptors in axon-myelin interactions. In particular, MAG is known to bind to some gangliosides on axons, including GD1a, GT1b, GT1β, and GQ1bα [1,25,26]. GM2/GD2 synthase-deficient mice lack complex gangliosides, including these gangliosides and exhibit similar phenotypes to *MAG*-deficient mice with regard to axon-myelin interactions relating to axon cytoarchitecture, axon stability, and axon regeneration [2–4]. On the other hand, our TEM analysis showed that the abundant small axons in the spinal cords of DKO mice lack myelin sheaths and that some of the large axons have thinner or less packed myelin sheaths, as well as lower axon circularity. Furthermore, MAG, MOG, and PLP signals were markedly reduced and rarely co-localized with NF in the cerebral cortices of DKO mice, suggesting that these myelin-associated proteins could not wrap around axons. MAG accumulated in oligodendrocyte cell bodies and was not distributed in myelin sheaths in DKO mice, probably because MAG-binding gangliosides were completely deficient in the axons of these mice. These phenotypes were more severe than those seen in *MAG*-deficient mice and quite similar to those observed in *MAG* and *PLP* double-deficient mice [27]. Since DKO mice lacked other gangliosides, such as GM3 and GD3, axon-myelin interactions might have been more severely impaired in our DKO mice than in GM2/GD2 synthase-deficient mice. The phenotype observed in DKO mice was rather similar to that of GM2/GD2 and GM3 synthase DKO mice and *Ugcg* cKO mice.

### Impaired neuronal maturation and PNN formation in DKO mice

Neuronal cells from DKO mice could adhere to collagen IV and fibronectin similar to wild-type cells, but their adhesion to laminin was much reduced. Furthermore, the relative levels of pLyn (Y396ph)/Lyn were significantly reduced in neurospheres from DKO mice than in wild-type neurospheres, while pTrkA (Y490ph)/TrkA levels also tended to be lower in DKO neurospheres. Laminin is reported to bind directly to GM1a and induces clustering of GM1a, TrkA, β1 integrin, and signaling molecules, such as Lyn, to activate neurite outgrowth in lipid rafts [1] [22]. Thus, the interaction of laminin with GM1a is important for the growth signal of nerve growth factor (NGF). Our results suggest that the reduction of GM1a-laminin interaction affects the NGF-TrkA-Lyn signaling cascade in DKO mice, resulting in reduced neurite outgrowth in neuronal cells from these mice. These defects might bring about an abundance of premature neurons in the cerebral cortex of DKO mice. Indeed, Tbr1 immunostaining indicated the presence of immature neurons in the upper layers of DKO cerebral cortices. Although laminin and gangliosides are not major components of PNNs [28], deficiencies in their interaction might also result in the impaired formation of PNNs in DKO mice. Taken together, our results suggest that the interaction between laminin and gangliosides is involved in neuronal maturation and PNN formation. Although both GM2/GD2 synthase-deficient mice and GM3 synthase-deficient mice cannot synthesize GM1a, the neuronal phenotypes of these mice are much milder than those of our DKO mice [2,3,5]. Thus, other gangliosides might compensate for GM1a loss and interact with laminin. Indeed, although laminin binds most strongly with GM1a, it can also bind with other gangliosides [22,29].

In conclusion, we have presented pivotal roles for gangliosides in the CNS, including neuronal maturation, PNN formation, and axonal and myelin formation using *B4galt5* and *B4galt6 D*KO mice, which completely lack LacCer synthase activity in the CNS. However, we could not identify the individual gangliosides that function in these neuronal processes, since DKO mice lacked all gangliosides. Further studies are needed to identify functions of individual gangliosides in the CNS. In this regard, we plan to perform a complementary study to treat neuronal cell cultures from DKO mice with candidate gangliosides and to then inject these gangliosides into DKO mice in an effort to rescue the neuronal deficits observed in these mice.

## Materials and methods

### Ethics statement

The animal experiments were conducted per the Fundamental Guidelines for Proper Conduct of Animal Experiment and Related Activities in Academic Research Institutions under the jurisdiction of the Ministry of Education, Culture, Sports, Science and Technology of Japan approved by the Committee on Animal Experimentation of Kanazawa University (AP-143169) and Kyoto University (MedKyo17013), Japan, and to the safety guidelines for gene manipulation experiments at Kanazawa University and Kyoto University.

For the preparation of brain section and tissue culture, animals were deeply anesthetized with a combined anesthetic (0.3 mg/kg of medetomidine, 4.0 mg/kg of midazolam, and 5.0 mg/kg of butorphanol).

### Generation of *B4galt5* cKO and *B4galt5/6* DKO mice

The generation of *B4galt5* KO (null) mice was described previously [16]. Briefly, the *B4galt5* gene was targeted by a vector to generate the *B4galt5*^*f(neo)*^ allele in embryonic stem (ES) cells. The neo cassette flanked by frt sites was removed by Flpe recombinase to generate the *B4galt5*^*flox*^ allele (S2 Fig). ES cells harboring the *B4galt5*^*f(neo)*^ allele were electroporated with an Flpe expressing plasmid (*pCAGGSFlpeIRESpuro*) [30] and a puromycin-resistant gene expressing plasmid (*pPGKpuro*), and puromycin-resistant colonies were transiently selected. Puromycin-resistant colonies were picked, and the desired ES clones were screened by PCR. Chimeric mice were generated from ES clones harboring the *B4galt5*^*flox*^ allele by the aggregation method [31] and mated with C57BL/6 mice to generate *B4galt5*^*flox*^ mice. The *B4galt5*^*flox*^ mice were backcrossed with C57BL/6 mice for five generations by the speed congenic method, as described previously [32] (http://shigen.nig.ac.jp/mouse/mmdbj/top.jsp). The resulting *B4galt5*^*flox*^ mice had 98.9% of their C57BL/6 alleles replaced. *B4galt5*^*flox*^ mice on a C57BL/6 background were crossed with *Nestin-Cre* Tg (C57BL/6) mice to generate *B4galt5* cKO mice. The generation of *B4galt6* KO mice was described previously [20]. We crossed *B4galt6* KO mice on a C57BL/6 background with *B4galt5*^*flox*^ mice and *Nestin-Cre* Tg mice to produce *B4galt5/6* DKO mice in the CNS.

### Genotyping by PCR

To determine the genotypes of mutant mice, genomic DNA was prepared from mouse tails and PCR analysis was conducted using the primers listed in S1 Table. For the *B4galt5* allele, the PCR products of the wild-type allele (193 bp) and flox allele (270 bp) were amplified using the same GT5 primer 1 and GT5 primer 2, as described previously [16]. A PCR product for the *B4galt5* null allele (350 bp) was amplified using the GT5 primer 1 and GT5 cko R2 primers. For the *B4galt6* allele, PCR products of the wild-type allele (290 bp) and mutant allele (550 bp) were amplified using the T6KO55/T6KO31 primers and Se1/T6KO31 primers, respectively. For the *Nestin-Cre* transgene, a 650-base pair (bp) PCR product to detect the *Cre* gene was amplified using Cre-F and Cre-R primers.

### qRT-PCR analysis

The expression levels of *B4galt5*, *B4galt6*, and *Ugcg* were measured using qRT-PCR. Total RNA was prepared from juvenile whole brains and 10 sub-regions of adult brains using an RNeasy Lipid tissue mini kit (Qiagen, Tokyo, Japan), and cDNA was reverse-transcribed using Prime Script RT Master Mix (TAKARA BIO, Shiga, Japan). qRT-PCR was performed using a Thermal Cycler Dice Real Time System Single (TAKARA BIO) with SYBR green reagent (SYBR Premix Ex Taq II; TAKARA BIO), according to the manufacturer’s instructions. The primers used for *B4galt5*, *B4galt6*, *Ugcg*, and *glyceraldehyde 3-phosphate dehydrogenase* (*GAPDH*) are listed in S1 Table. The PCR conditions were as follows: 94°C for 5 min, followed by 40 cycles of 94°C for 15 s, 60°C for 30 s, and a dissociation protocol was also performed. The mRNA levels of the targets were calculated and normalized to the *GAPDH* mRNA levels.

### Measurement of GlcCer and LacCer synthase activity

All steps were performed at 0–4°C in solutions containing protease inhibitors (cOmplete, Mini, EDTA-free, Roche, Basel, Switzerland). Brain hemispheres were homogenized in 1 mL of 0.25 M sucrose using a dounce homogenizer. The lysates (0.5 mL) were added to 0.5 mL of 0.25 M sucrose and 1 mL of 5 mM HEPES (pH 7.3)/0.34 M sucrose and centrifuged (7,000 × *g* for 10 min). The pellets were dissolved in 0.5 mL of 0.25 M sucrose and 0.5 mL of 5 mM HEPES (pH 7.3)/0.34 M sucrose and centrifuged (7,000 × *g* for 10 min). These supernatants (1 mL combined after the 1^st^ and 2^nd^ centrifugation) were mixed with 1 mL of 0.25 M sucrose and 1 mL of 5 mM HEPES (pH 7.3)/0.34 M sucrose and centrifuged at 100,000 × *g* for 60 min. The pellets were resuspended in 0.2 mL of 50 mM HEPES (pH 7.3)/5% glycerol and sonicated for 15 s for the preparation of membrane fractions.

The activities of GlcCer and LacCer synthases were measured, as previously described [33]. For the GlcCer synthase assay, 50 μL of a reaction mixture containing 50 pmol of C6-NDB-Cer as the liposome, 500 μM UDP-Glc, 1 mM ethylenediaminetetraacetic acid (EDTA), and 10 μL of the membrane fraction in 25 mM Tris-HCl, pH7.5, was incubated at 37°C for 90 min. For the LacCer synthase assay, 50 μL of a reaction mixture containing 50 pmol C6-NDB-GlcCer as the liposome, 200 μM UDP-Gal, 5 mM MgCl_2_, 5 mM MnCl_2_, 5 mM gluconic acid δ-lactone, and 10 μL of the membrane fraction in 50 mM HEPES, pH 7.3, was incubated at 37°C for 20 min. The reaction was stopped by the addition of 200 μL of chloroform/methanol (2/1, v/v). The lower phase was withdrawn after centrifugation of the sample at 16,000 × *g* for 3 min, dried, and dissolved in 200 μL of hexane/2-propanol/H_2_O (45/54/1, v/v/v). After centrifugation at 16,000 × *g* for 5 min, 50 μL of supernatants were injected into the HPLC column (Intertosil SIL 150A-5, 4.6 × 250 mm, GL Sciences, Japan) and eluted at a flow rate of 1.5 mL/min with 2-propanol/hexane/H_2_O (54/45/1, v/v/v), for the GlcCer synthase assay, or 2-propanol/hexane/H_2_O/phosphoric acid (110/84/5.9/0.1, v/v/v/v), for the LacCer synthase assay. Fluorescence was determined using a fluorescence detector (F-1050, Hitachi, Tokyo, Japan) set to excitation and emission wavelengths of 470 nm and 530 nm, respectively.

### Analysis of glycosphingolipids

Brain lysates (200 μL of 10 mg/mL) were mixed with 1.2 mL of methanol and 2 mL of chloroform and incubated at 37°C for 1 h with shaking. After centrifugation at 1,000 × *g* for 10 min, the pellets were mixed with chloroform/methanol/H_2_O (1/2/0.8, v/v/v), incubated at 37°C for 2 h with shaking and centrifuged again at 1,000 × *g* for 10 min. The supernatants from the 1^st^ and 2^nd^ centrifugation were mixed, dried, and dissolved in 2 mL of methanol. The extracted GSLs (2 mL) were mixed with 25 μL of 4 M NaOH and incubated at 37°C for 2 h. The GSLs were mixed with 25 μL of 4 M acetic acid and 2 mL of H_2_O, and applied to an OASIS HLB cartridge (normal-phase cartridge, Nihon Waters, Tokyo, Japan). The cartridge was washed with 1 mL of methanol/H_2_O (1/1, v/v) and 1 mL of H_2_O, and GSLs were eluted with 1 mL of methanol. GSLs were dried, dissolved in 30 μL of chloroform/methanol (1/2, v/v), and applied to the high performance thin layer chromatography (HPTLC) plate, which was developed with chloroform/methanol/0.02% CaCl_2_ (5/4/1, v/v). The TLC plate was visualized using orcinol-H_2_SO_4_ reagent.

### Determination of GlcCer and GalCer amounts

The amounts of GlcCer and GalCer were determined using normal-phased HPLC after derivatization with o-phthalaldehyde as described previously [34], with minor modifications. Here, α-mannosylceramide was used as an internal standard instead of C6-NBD-GlcCer.

### Lipid extraction for LC-ESI MS analysis

For extraction of sphingolipids, 100 μL of mouse brain lysates (1 mg of protein) was mixed with 600μL of chloroform/methanol (1:2, v/v) containing 0.25 μM Ceramide/Sphingoid Internal Standard Mixture I (Avanti Polar Lipids, Alabaster, AL, USA) and incubated at 37°C for 14 h. After incubation, 50 μL of 1 M potassium hydroxide in MeOH were added, and the mixture was incubated at 37°C for 120 min, followed by addition of 50 μL of 1 M acetic acid, 300 μL of distilled water, and 250 μL of chloroform. After vortexing for 30 s, the mixture was centrifuged at 10,000 × *g* for 5 min, and the upper and middle phases were removed. The lower phase was transferred to a new tube and dried up by Speed Vac. The mixture was dissolved in 110 μL of 2-propanol followed by centrifugation at 10,000 × *g* for 5 min. The 100 μL of each sample was transferred into a new glass vial and 5 μL were injected into the LC-MS system.

### LC-ESI MS analysis

The analysis of sphingolipids was performed by LC-ESI MS, as described previously [35] with adjustments. LC-ESI MS was performed by an HPLC system (Agilent Technologies) combined into an MS appliance (3200 QTRAP LC/MS/MS; AB Sciex). Sphingolipids were separated by reverse-phase chromatography in a binary solvent gradient with a flow rate of 200 μL/min, using an InertSustain C18 column (2.1 mm × 150 mm, 5 μm; GL Sciences). The gradient was started with 3% B (2-propanol in 0.1% formic acid and 0.028% ammonium) in buffer A (acetonitrile/methanol/distilled water, 19:19:2, v/v/v containing 0.1% formic acid and 0.028% ammonium) and was held for 3 min. Afterwards the gradient was converted to 40% B for 21 min, 70% B for 1 min, and was held for 7 min. The gradient was turned back to the initial conditions for 1 min and was held for 7 min for re-equilibration.

For SM analysis, precursor ion scan at *m*/*z* 184, corresponding to the phosphocholine head group, was applied to determine the molecular species of SM. Precursor ion scan at *m*/*z* 264, corresponding to Sph (d18:1), and at *m*/*z* 266, corresponding to DHS (d18:0), were devoted to determine the molecular species of Cer. Free Sph (d18:1) and DHS (d18:0) were detected by the single dehydration product at *m/z* 282 and *m/z* 284, respectively [36]. Finally, SM, Cer, and Sph were quantified by using multiple reaction monitoring (MRM), as shown in S3 Table.

### Histological procedures

Mice at postnatal day 19 to 21 were deeply anesthetized and perfused with phosphate buffered saline (PBS) followed by 4% paraformaldehyde in PBS. The brains and spinal cords were post-fixed overnight and then sunk in 30% sucrose solution or embedded in paraffin. Cryo- or paraffin sections were cut sagittally or coronally. For the preparation of spinal cord samples, Technovit 7100 (Kulzer, Wehrheim, Germany) was used according to the manufacturer’s instruction. Kluver-Barrera's staining was performed using 0.1% Luxol Fast Blue and 0.1% cresyl violet solutions, and broad morphological features were observed under a microscope (IX-71, Olympus, Tokyo, Japan).

### Immunohistochemistry

Immunohistochemistry was carried out to investigate the morphological features of neural cells, the numbers of each cell type, and their glycosylation status. The primary antibodies used were as follows: anti-beta III tubulin (ab78078; Abcam, Cambridge, USA), anti-160 kDa NF medium (ab7794; Abcam), anti-MBP (ab626931; Abcam), anti-MAG (9043S CST, Danvers, USA), anti-MOG (ab32760; Abcam), anti-PLP (ab28486; Abcam), anti-APC (OP80; Merck), anti-Tbr1 (ab31940; Abcam), and fluorescein isothiocyanate (FITC)-conjugated WFA (Vector Laboratories, Burlingame, USA). The sections were incubated with primary antibodies (1/200 dilution) overnight at 4°C, rinsed three times in PBS, and incubated with Alexa Fluor-488 or Alexa Fluor-546 secondary antibodies (1/200 dilution) for 2 h at room temperature. The stained sections were sealed using cover slips and ProLong Diamond antifade mountant with 4',6-diamidino-2-phenylindole (DAPI; Thermo Fisher Scientific, Waltham, USA). Immunofluorescence signals were observed and evaluated under a fluorescence microscope (BZ-X710, Keyence, Osaka, Japan). Representative immunohistochemistry images from 2–3 different mice per genotype are shown in Figs 4, 5, 6, 7 and 8.

### Transmission electron microscope (TEM) analysis

TEM analysis was performed as described previously [37]. Anesthetized mice were fixed with a 2.5% glutaraldehyde and 2% paraformaldehyde solution in 0.1 M phosphate buffer (PB) at 4°C. After perfusion, 1 mm-thick parasagittal sections of the cerebral hemispheres and coronal sections of the spinal cords at the cervical to superior thoracic level were immersed in the same fixative before use at 4°C. The thick sections were post-fixed in 1% osmium tetroxide in 0.1 M PB, dehydrated, and embedded in epoxy resin; then ultra-thin sections were cut at 100 μm. To observe the dorsal cerebral cortex at bregma and the distribution of myelinated axons in, and anterior and lateral to the corticospinal tracts in the spinal cord, ultra-thin sections were examined under TEM (EM-002B; Topcon, Tokyo, Japan) at a low (× 500) and high magnification (× 2500). The axon circularity and distance between nuclei were calculated using ImageJ (NIH, Bethesda, USA).

### Neurosphere assay

#### Cell preparation and proliferation assay

We employed a neurosphere assay to evaluate cell proliferation and differentiation of neural stem cells. For neurosphere preparations, the GE was dissected from the telencephalon of E14.5 embryos. The GE was incubated with Accutase (Innovative Cell Technologies, San Diego, USA) in a 5% CO_2_ incubator at 37°C. Single cells were collected and incubated in neurosphere medium at a cell count of 2 × 10^5^ cells/mL. The culture medium contained the following factors in Neurobasal Medium (Thermo Fisher Scientific, Waltham, USA): 1% GlutaMax (Thermo Fisher Scientific), 1% PenStrep (Thermo Fisher Scientific), 2% B-27 supplement (Thermo Fisher Scientific), 20 ng/mL fibroblast growth factor 2 (PeproTech, Rocky Hill, USA), and 20 ng/mL epidermal growth factor (PeproTech). To maintain cell growth, half of the amount of the neurosphere medium was added fresh on the fourth day post-plating. On the seventh day post-plating, floating neurospheres were collected and dissociated using Accutase, as described above, and the cells were plated for the next passage culture. These procedures continued until the cells were cultured to their tertiary stage. We used ultra-low attachment plates (Costar#3473, Corning, USA) for the proliferation assay and low adhesion plates (MS-8006R, Sumitomo Bakelite, Tokyo, Japan) for the differentiation assay.

### Cell differentiation assay

The differentiation assay was conducted using tertiary cultured cells. Cells were cultured in a cell culture slide chamber (SPL-30108, SPL Life Science, Pocheon, Korea) pretreated with several ECMs, including laminin (5, 10, 20 mg/mL; 354232, Corning), fibronectin (5, 10, 20 mg/mL; F4759, Sigma, St. Louis, USA), and collagen IV (5, 10, 20 mg/mL; 3440-010-01, Trevigen, Gaithersburg, USA). For the differentiation assay, the culture medium contained the following factors in Neurobasal Medium: 1% GlutaMax, 1% PenStrep, 2% B-27 supplement, and 1% fetal bovine serum (Thermo Fisher Scientific, 10437). Ten days after plating, the number of cells attached to the slide and neuronal morphological features were observed following the immunostaining procedure.

### Western blot analysis

Neurospheres cultured on 5 μg/mL laminin-coated glass chambers for differentiation were lysed in NP-40 buffer (150 mM NaCl, 50 mM Tris-Cl, 1% NP-40), containing a phosphatase inhibitor cocktail (Nacalai, Kyoto, Japan), and sonicated with Bioruptor (Diagenode, Liege, Belgium). The samples were applied on 5–12% SuperSep SDS-PAGE gel (Wako, Osaka, Japan) and transferred to PVDF membrane (Merck Millipore, Darmstadt, Germany). The membranes were incubated overnight with primary antibodies in Can Get Signal immunoreaction enhancer solution (Toyobo, Osaka, Japan) at 4°C. After the membranes were treated with secondary antibody at room temperature for 1 h (for all antibodies) and streptavidin-poly HRP (Thermo Scientific) (only for anti-TrkA and anti-TrkA Y490ph), the protein bands were detected using ImmunoStar LD (Wako). Antibodies and dilutions used in western blotting were as follows: rabbit anti-TrkA Y490ph (1:500; CST, MA, USA, 9141s), rabbit anti-TrkA (1:500; Abcam, Cambridge, UK, 76291), rabbit anti-Lyn Y396ph (1:2000; Abcam, 40660), rabbit anti-Lyn (1:500; CST, 2796s), goat anti-rabbit IgG-HRP (1:2000; Abcam, 97023), goat anti-rabbit IgG-biotin (1:1000; Vector, CA, USA, BA-1000), and Pierce streptavidin poly-HRP (1:3000; Thermo Scientific, MA, USA, 21140).

Western blotting for brain homogenates was carried out, as described previously [38], using total brain proteins, extracted using total protein extraction kit (P501S; 101 Bio, AC, USA). Primary and secondary antibodies were as follows. anti-MBP (1:5000; ab626931; Abcam), anti-MAG (1:5000; 9043S CST, Danvers, USA), anti-MOG (1:5000; ab32760; Abcam), anti-PLP (1:5000; ab28486; Abcam), anti-β-actin (1:5000; bs-0061R, Bioss, MA, USA), and goat anti-rabbit IgG-HRP (1:100,000; Abcam, 97023).

### Inhibitor assay for protein synthesis

Neurospheres (1x10^4^ cells/100 μL) plated on 96-well plastic plates were incubated for 1 or 2 h in neurosphere medium containing cycloheximide (ab120093; Abcam) at 0, 10, 20 or 40 μg/mL. The cells were stained using trypan blue (29853–42; Nakarai, Kyoto, Japan), and the number of cells stained by trypan blue was counted as dead cells.

### Statistical analysis

Differences were evaluated using a Student’s t-test. Differences with a two-sided *P* value <0.05 were deemed statistically significant. The results are expressed as means ± standard deviation (SD).

## Supporting information

S1 Fig Biosynthetic pathway of sphingolipids and glycosphingolipids (GSLs), including gangliosides. Various GSLs, including gangliosides, are synthesized from ceramide (Cer). Sphingolipids associated with Cer, including sphingomyelin (SM), sphingosine (Sph) and dihydrosphingosine (DHS) are also shown. GlcCer synthase, LacCer synthase, GM3 synthase and GM2/GD2 synthase are indicated. GM1a, GD1a, GD1b and GT1b, shown by blue shadow, are abundant gangliosides in the brain.
(TIF)

S2 Fig Targeting strategy for the generation of *B4galt5* conditional knockout (cKO) mice. *B4galt5*^*wt*^: wild-type *B4galt5* allele; targeting vector: a targeting vector to disrupt the *B4galt5* gene by homologous recombination; *B4galt5*^*f(neo)*^: *B4galt5*^*flox*^ allele containing the neo gene; *B4galt5*^*flox*^: *B4galt5*^*flox*^ allele after Flp recombinase treatment to remove the neo gene; *B4galt5*^*cKO*^: *B4galt5* cKO allele after breeding with Nestin-Cre mice to remove exons 4–7 of the *B4galt5* gene. neo, neo-resistant gene; DT-A, diphtheria toxin A fragment; closed boxes with numbers, exons of the *B4galt5* gene; blue triangles, loxP sites; red triangles, frt sites; orange arrows, primers 1 and 2 for genotyping; X, Xho I; P, Pvu II.
(TIF)

S3 Fig Expression of *B4galt5*, *B4galt6*, and *Ugcg* genes in brains analyzed by qRT-PCR. mRNA levels of *B4galt5* in postnatal mouse whole brains (A; n = 3 per each genotype, per stage) and in 10 adult brain sub-regions (B; n = 3 per each genotype). Blue bars, *B4galt5*^*flox*^ mice (control); red bars, *B4galt5* conditional knockout (cKO) mice. mRNA levels of *B4galt6* in postnatal mouse whole brains (C) and in 10 adult brain sub-regions (D). Blue bars, *B4galt5*^*flox*^ mice (control); red bars, *B4galt5* cKO mice. OB, olfactory bulb; Str, striatum; Cx, cerebral cortex; Tha, thalamus; Hyp, hypothalamus; Hip, hippocampus; Pn, pons; MB, midbrain; Ce, cerebellum; Md, medulla oblongata. (E) *Ugcg* mRNA levels in whole brains of wild-type (wt; n = 4) and double knockout (DKO; n = 2) mice at 3 weeks of age. Note that small squares on the bar graph indicate the individual values of each sample.
(TIF)

S4 Fig Body weights of mice with 4 different genotypes of *B4galt5* and *B4galt6* genes at 14 and 21 days of age. Body weights of *B4galt5*^*flox*^*/B4galt6* ht (n = 8), *B4galt5*^*flox*^*/B4galt6* knockout (KO) (n = 8), *B4galt5* conditional KO (cKO)/*B4galt6* ht (n = 8) and *B4galt5* cKO/*B4galt6* KO (n = 8) mice. *, *p*<0.01.
(TIF)

S5 Fig Ganglioside analysis by high performance thin layer chromatography (HPTLC). Glycosphingolipids (GSLs) extracted from brain homogenates from wild-type (wt), *B4galt5*^*flox*^ (GT5 flox), *B4galt5* conditional knockout (GT5 cKO), and *B4galt6* KO (GT6 KO) mice were separated by HPTLC. M1 and M2, standard GSLs indicated.
(TIF)

S6 Fig Western blot analysis of myelin-associated proteins. Western blot analysis of brain homogenates from wild-type (wt, n = 2) and double knockout (DKO; n = 2) mice at 3 weeks of age, using anti-MBP, MAG, MOG, PLP and β-actin antibodies. Molecular weight markers are indicated in the left. The expected molecular weights of each protein are shown in parenthesis.
(TIF)

S1 Table Primers used for genotyping and quantitative RT-PCR. (TIF)

S2 Table Detailed analysis of individual sphingolipid molecules including ceramide (Cer), sphingomyelin (SM), sphingosine (Sph) and dihydrosphingosine (DHS). (TIF)

S3 Table Multiple reaction monitoring (MRM) pairs of SM, Cer, and Sph in LC-ESI MS. (TIF)
