# Supplementary figures and images for: Lactosylceramide synthases encoded by B4galt5 and 6 genes are pivotal for neuronal generation and myelin formation in mice
Source: PLoS Genet. 2018 Aug 16;14(8):e1007545. doi: 10.1371/journal.pgen.1007545 (PMC6095488; doi:10.1371/journal.pgen.1007545)

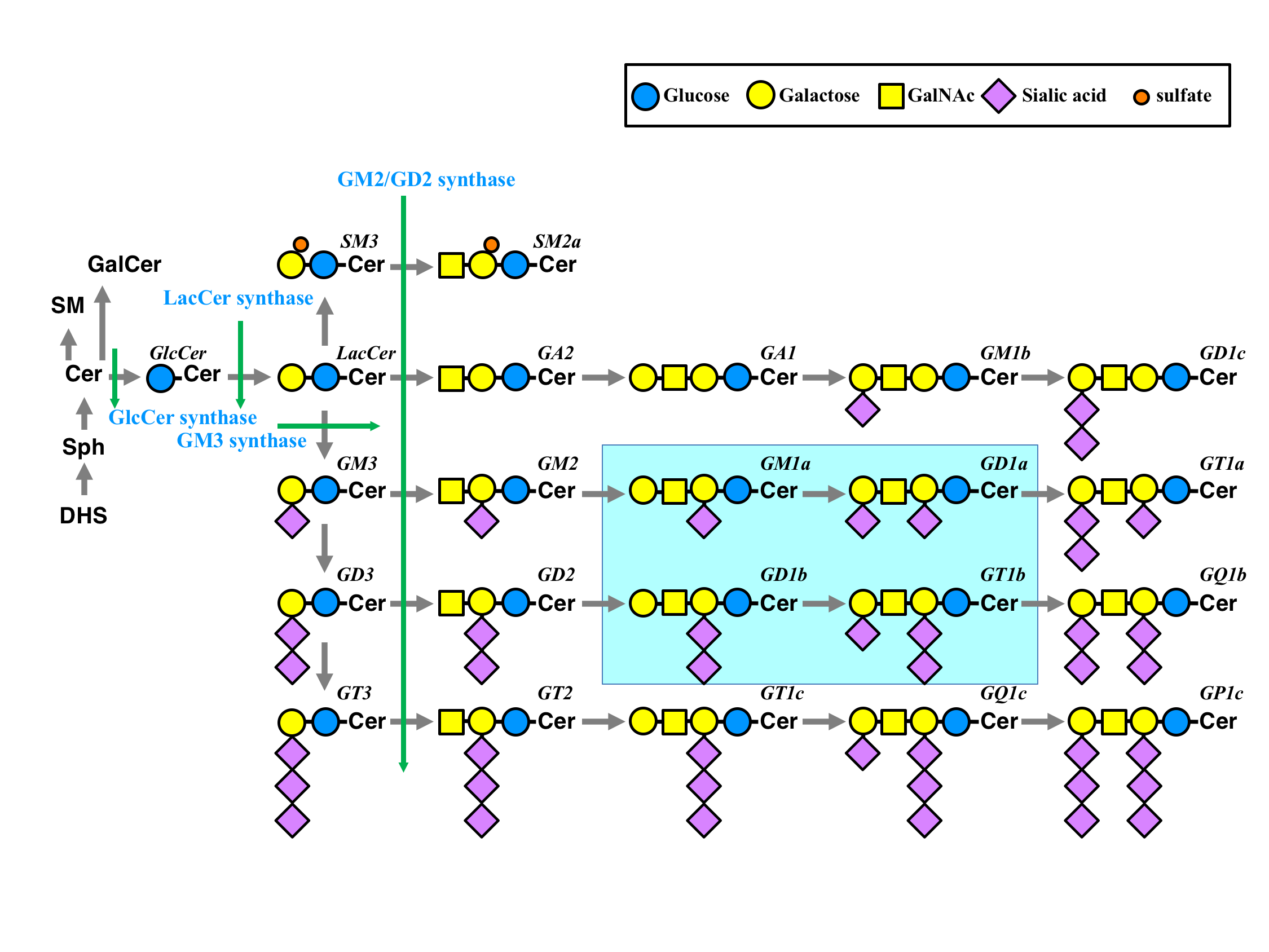

Supplement: S1 Fig — Various GSLs, including gangliosides, are synthesized from ceramide (Cer). Sphingolipids associated with Cer, including sphingomyelin (SM), sphingosine (Sph) and dihydrosphingosine (DHS) are also shown. GlcCer synthase, LacCer synthase, GM3 synthase and GM2/GD2 synthase are indicated. GM1a, GD1a, GD1b and GT1b, shown by blue shadow, are abundant gangliosides in the brain. (TIF) [file pgen.1007545.s001.tif]

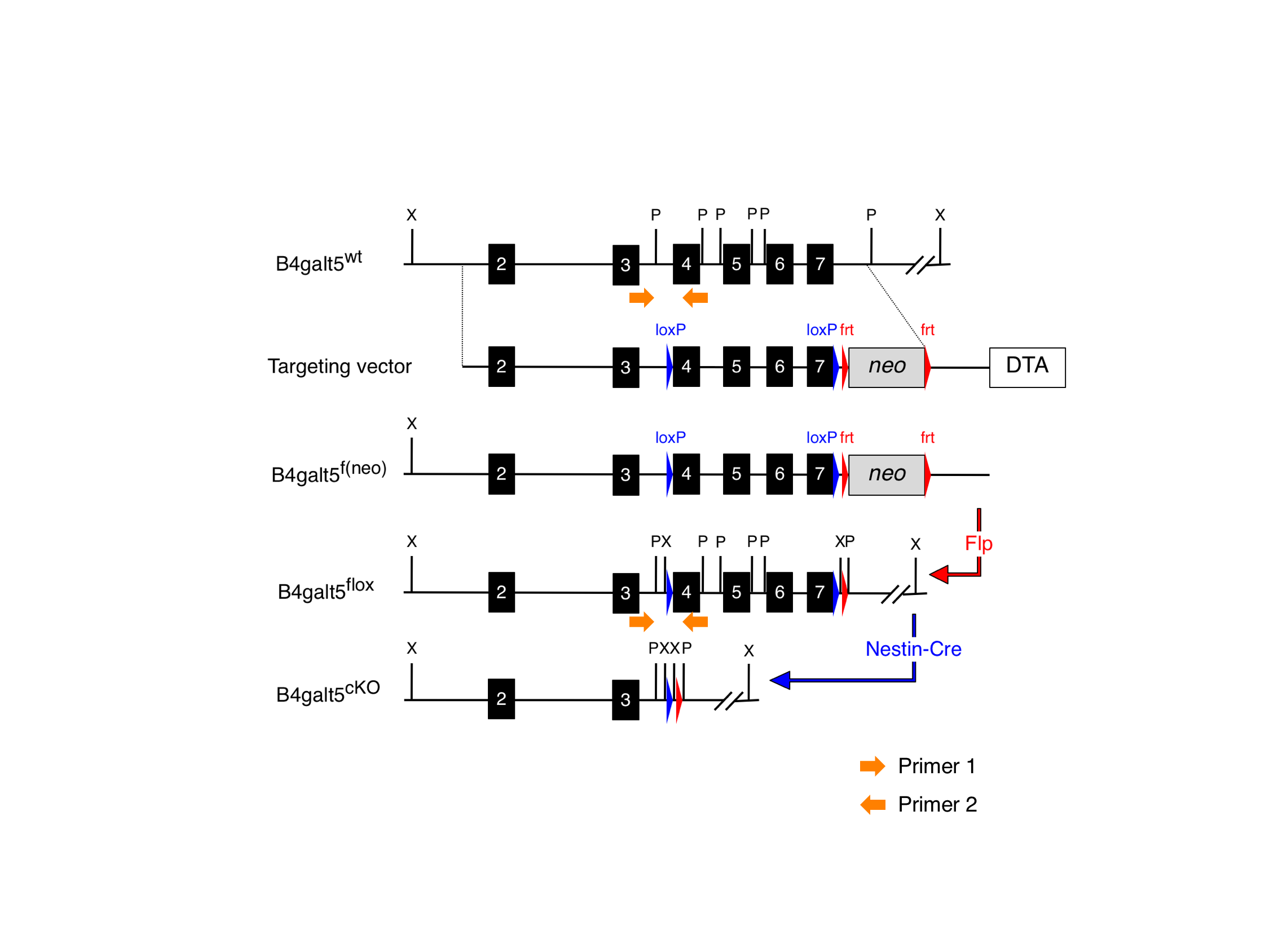

Supplement: S2 Fig — B4galt5wt: wild-type B4galt5 allele; targeting vector: a targeting vector to disrupt the B4galt5 gene by homologous recombination; B4galt5f(neo): B4galt5flox allele containing the neo gene; B4galt5flox: B4galt5flox allele after Flp recombinase treatment to remove the neo gene; B4galt5cKO: B4galt5 cKO allele after breeding with Nestin-Cre mice to remove exons 4–7 of the B4galt5 gene. neo, neo-resistant gene; DT-A, diphtheria toxin A fragment; closed boxes with numbers, exons of the B4galt5 gene; blue triangles, loxP sites; red triangles, frt sites; orange arrows, primers 1 and 2 for genotyping; X, Xho I; P, Pvu II. (TIF) [file pgen.1007545.s002.tif]

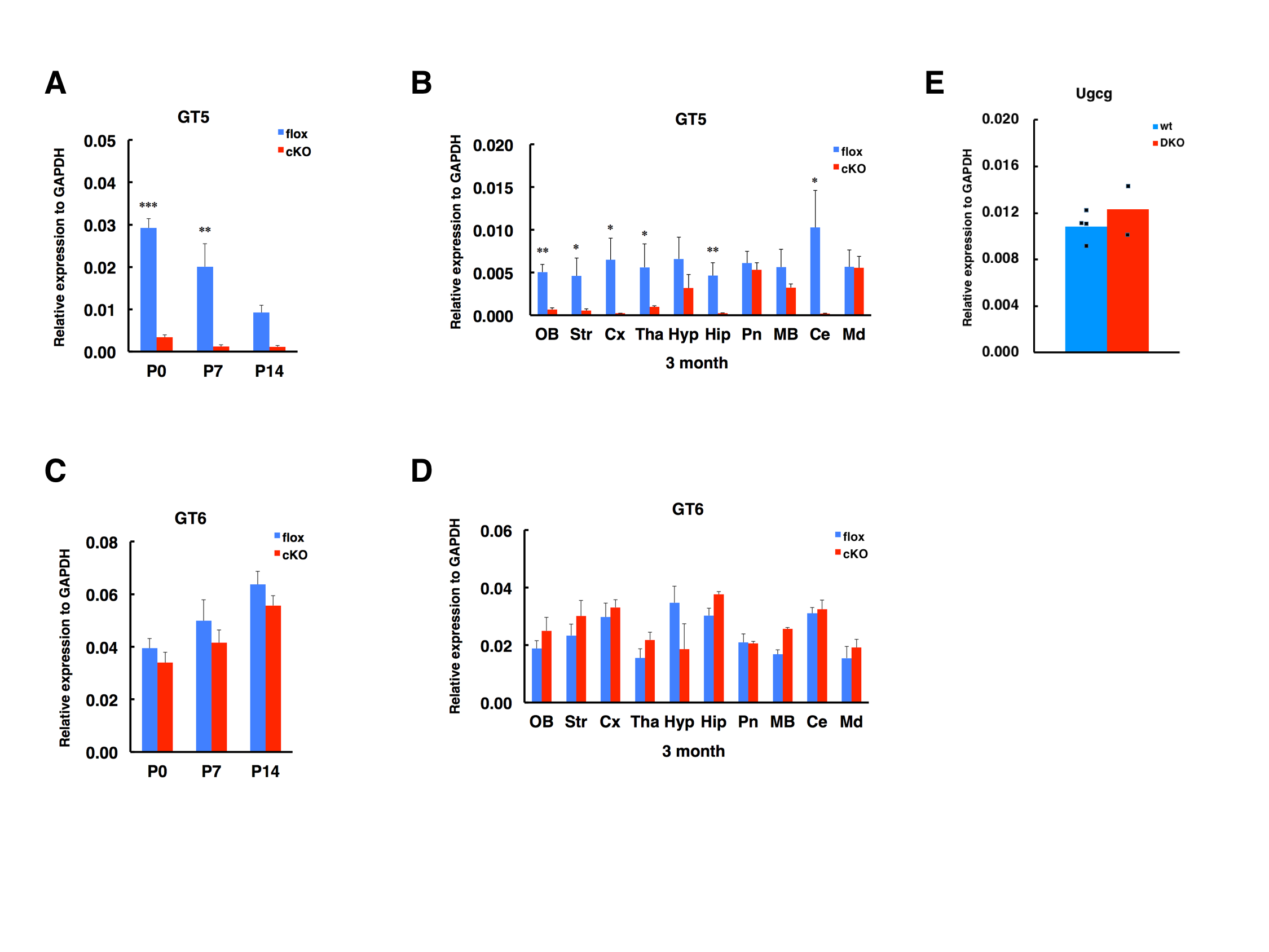

Supplement: S3 Fig — mRNA levels of B4galt5 in postnatal mouse whole brains (A; n = 3 per each genotype, per stage) and in 10 adult brain sub-regions (B; n = 3 per each genotype). Blue bars, B4galt5flox mice (control); red bars, B4galt5 conditional knockout (cKO) mice. mRNA levels of B4galt6 in postnatal mouse whole brains (C) and in 10 adult brain sub-regions (D). Blue bars, B4galt5flox mice (control); red bars, B4galt5 cKO mice. OB, olfactory bulb; Str, striatum; Cx, cerebral cortex; Tha, thalamus; Hyp, hypothalamus; Hip, hippocampus; Pn, pons; MB, midbrain; Ce, cerebellum; Md, medulla oblongata. (E) Ugcg mRNA levels in whole brains of wild-type (wt; n = 4) and double knockout (DKO; n = 2) mice at 3 weeks of age. Note that small squares on the bar graph indicate the individual values of each sample. (TIF) [file pgen.1007545.s003.tif]

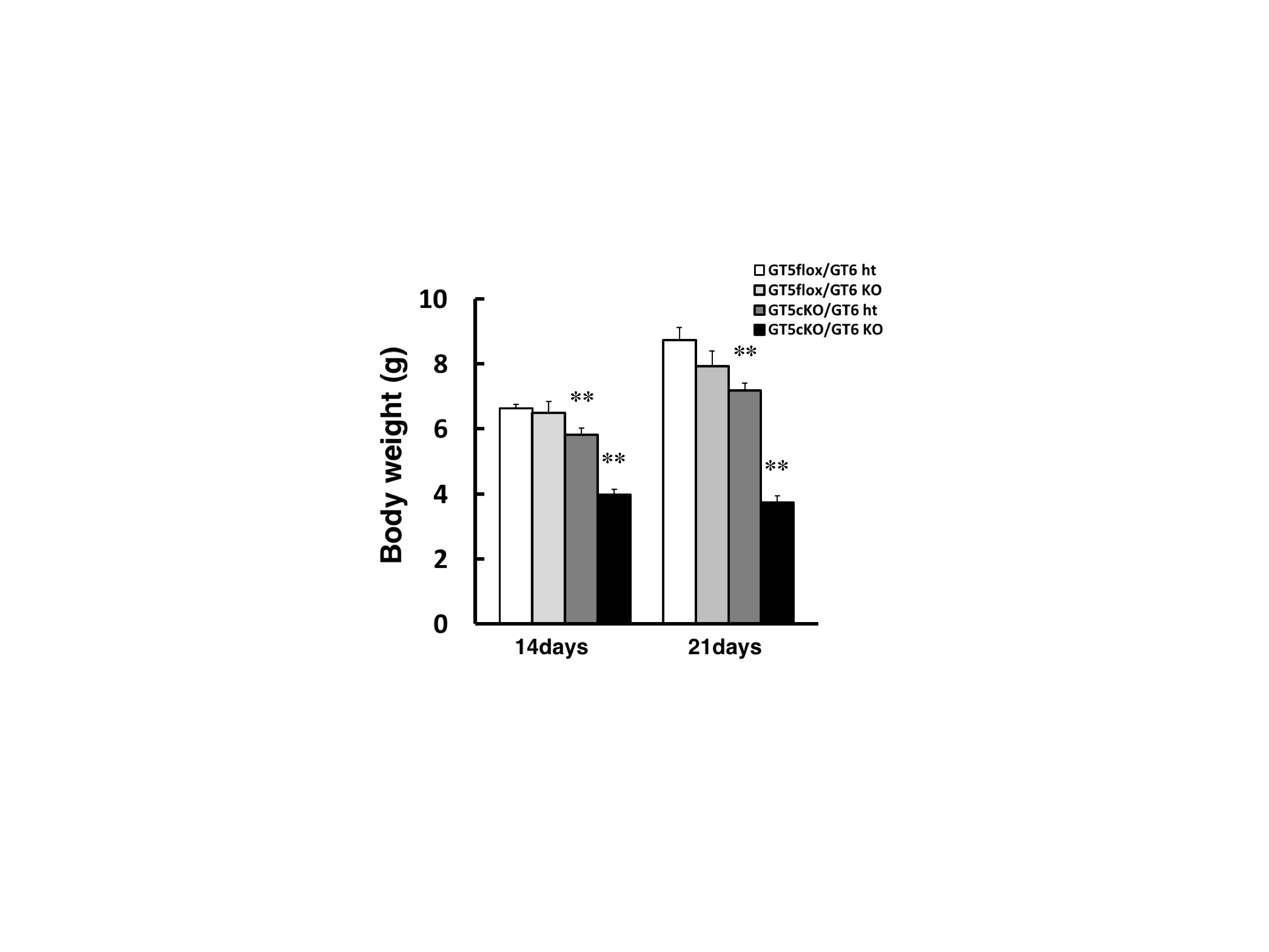

Supplement: S4 Fig — Body weights of B4galt5flox/B4galt6 ht (n = 8), B4galt5flox/B4galt6 knockout (KO) (n = 8), B4galt5 conditional KO (cKO)/B4galt6 ht (n = 8) and B4galt5 cKO/B4galt6 KO (n = 8) mice. *, p<0.01. (TIF) [file pgen.1007545.s004.tif]

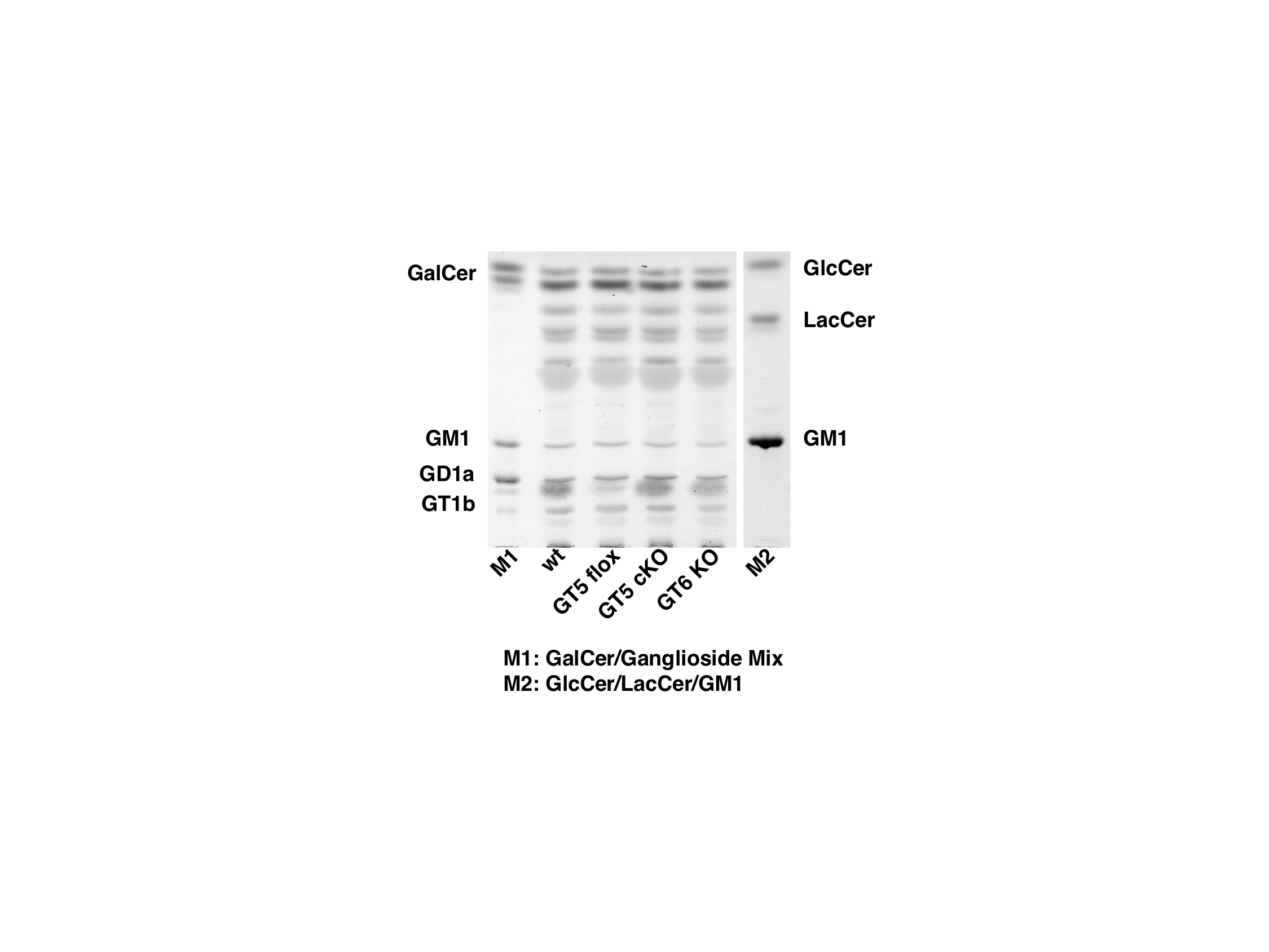

Supplement: S5 Fig — Glycosphingolipids (GSLs) extracted from brain homogenates from wild-type (wt), B4galt5flox (GT5 flox), B4galt5 conditional knockout (GT5 cKO), and B4galt6 KO (GT6 KO) mice were separated by HPTLC. M1 and M2, standard GSLs indicated. (TIF) [file pgen.1007545.s005.tif]

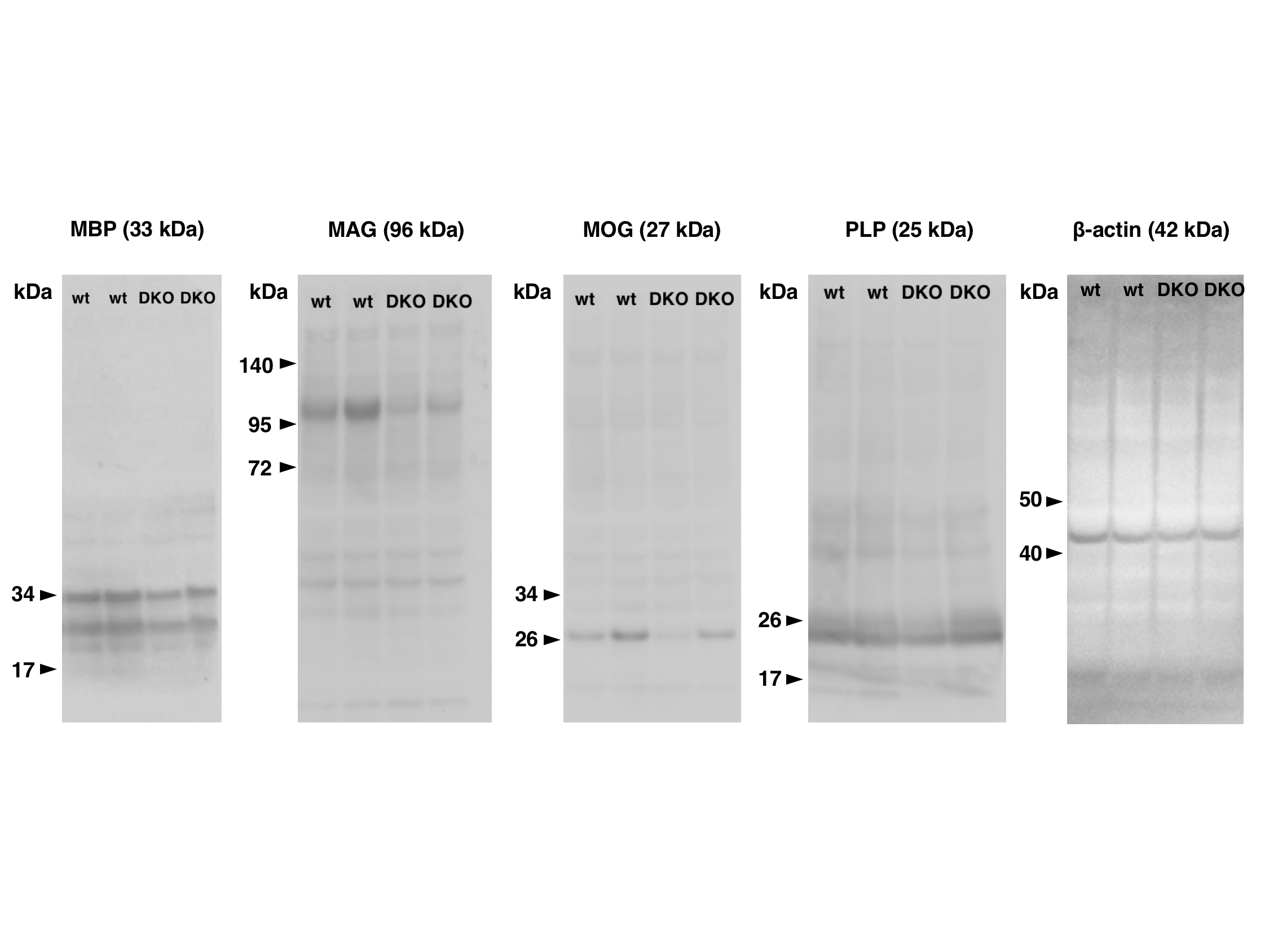

Supplement: S6 Fig — Western blot analysis of brain homogenates from wild-type (wt, n = 2) and double knockout (DKO; n = 2) mice at 3 weeks of age, using anti-MBP, MAG, MOG, PLP and β-actin antibodies. Molecular weight markers are indicated in the left. The expected molecular weights of each protein are shown in parenthesis. (TIF) [file pgen.1007545.s006.tif]

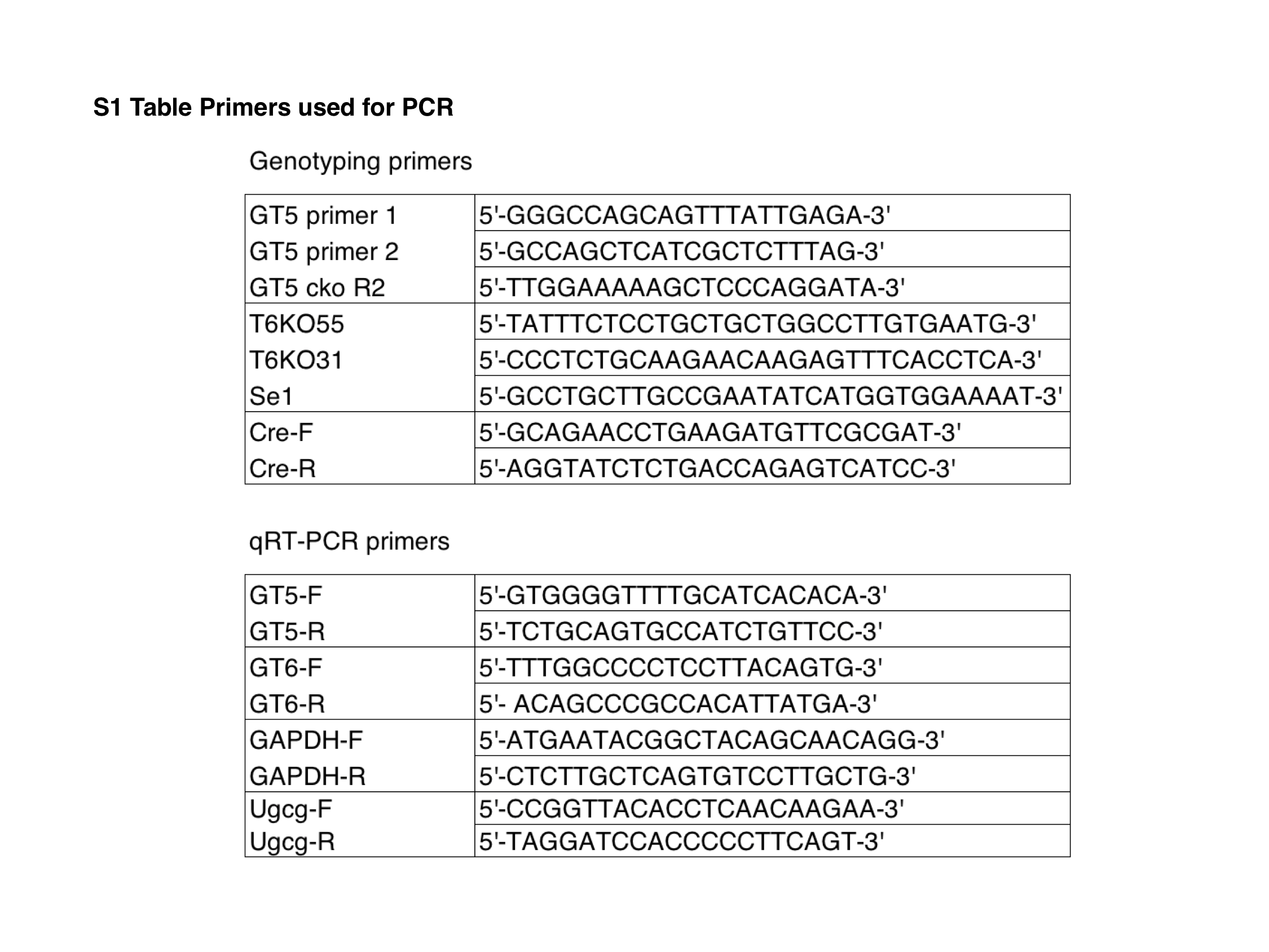

Supplement: S1 Table — (TIF) [file pgen.1007545.s007.tif]

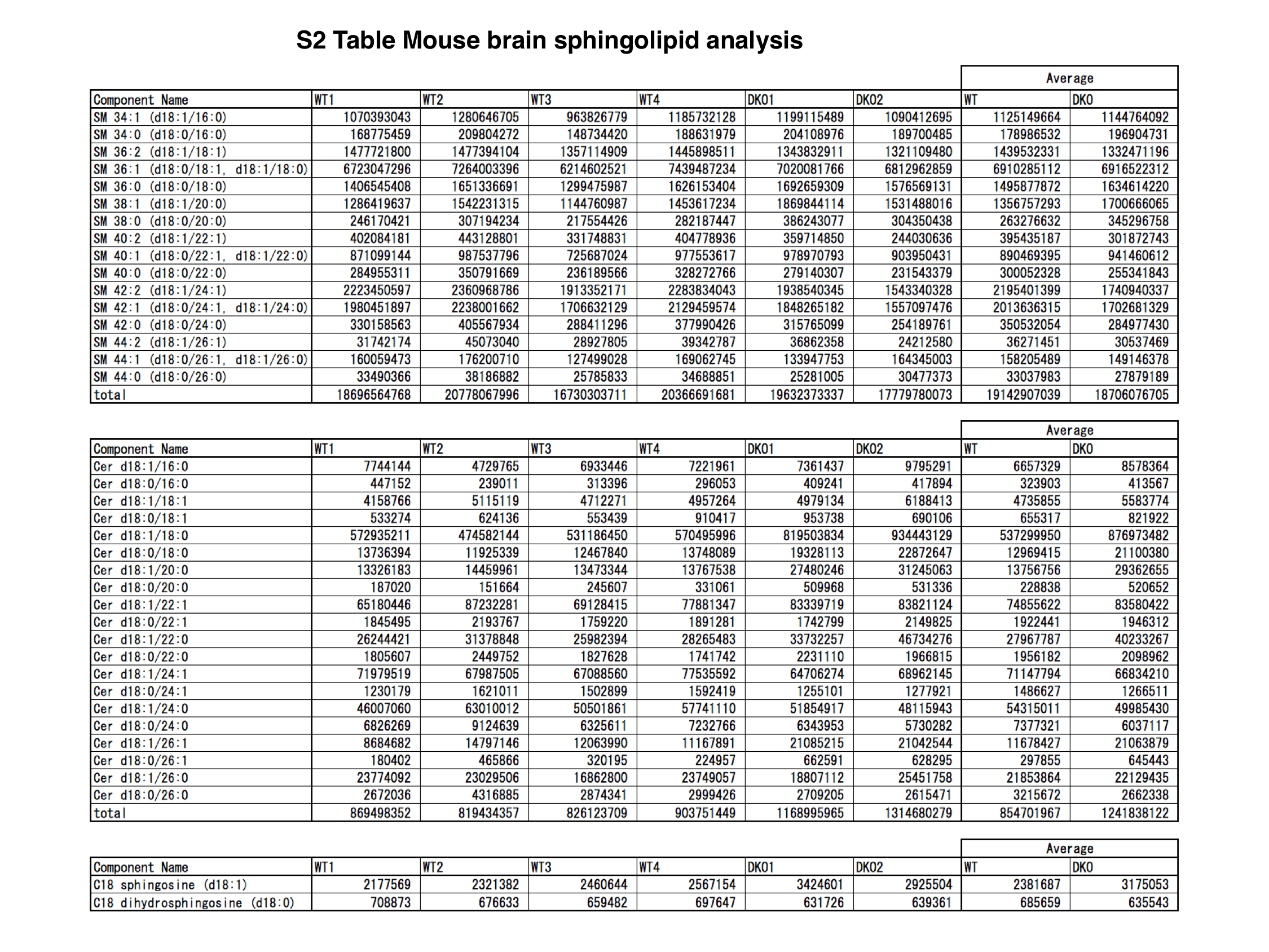

Supplement: S2 Table — (TIF) [file pgen.1007545.s008.tif]

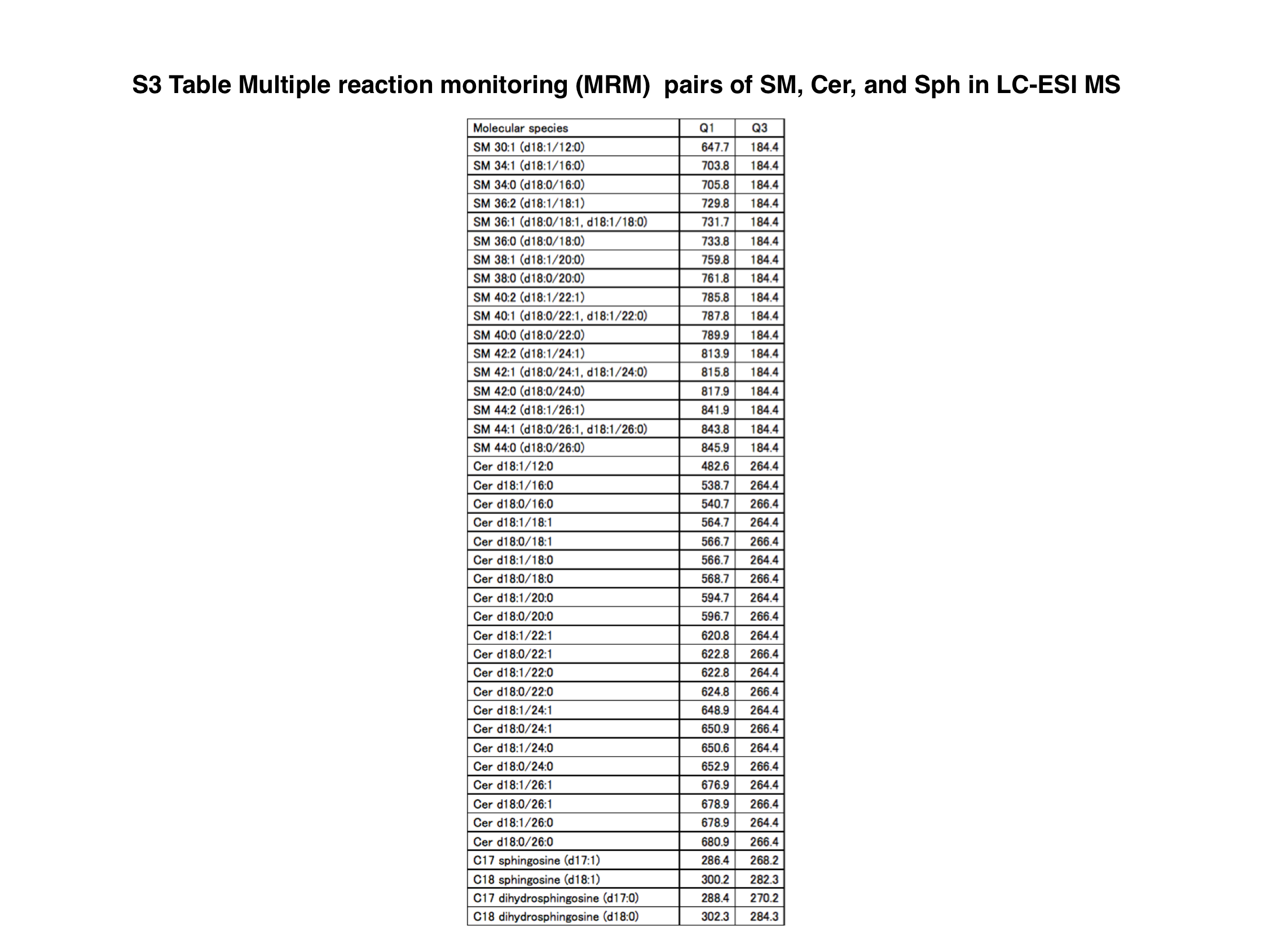

Supplement: S3 Table — (TIF) [file pgen.1007545.s009.tif]
